# Transcriptomic analysis reveals differences in the regulation of amino acid metabolism in asexual and sexual planarians

**DOI:** 10.1038/s41598-019-42025-z

**Published:** 2019-04-16

**Authors:** Kiyono Sekii, Shunta Yorimoto, Hikaru Okamoto, Nanna Nagao, Takanobu Maezawa, Yasuhisa Matsui, Katsushi Yamaguchi, Ryohei Furukawa, Shuji Shigenobu, Kazuya Kobayashi

**Affiliations:** 1 Department of Biology, Faculty of Agriculture and Life Science, Hirosaki University, 3 Bunkyo-cho, Hirosaki, Aomori 036-8561 Japan; 2 Department of Integrated Science and Technology, National Institute of Technology, Tsuyama College, 624-1 Numa, Tsuyama, Okayama 708–8509 Japan; 3 Cell Resource Center for Biomedical Research, Institute of Development, Aging and Cancer, Tohoku University, 4-1 Seiryo-machi, Sendai, 980-8575 Japan; 4 NIBB Core Research Facilities, National Institute for Basic Biology, 38 Nishigonaka Myodaiji, Okazaki, 444-8585 Japan; 5 Division of Biomedical Information Analysis, Iwate Tohoku Medical Megabank Organization, Iwate Medical University, 2-1-1 Nishitokuda, Yanaba-cho, Shiwa-gun, Iwate 028-3694 Japan; 6 Department of Biology, Research and Education Center for Natural Sciences, Keio University, 4-1-1 Hiyoshi, Kohoku-ku, Yokohama, Kanagawa 223-8521 Japan; 7 Department of Basic Biology, School of Life Science, SOKENDAI (The Graduate University for Advanced Studies), 38 Nishigonaka Myodaiji, Okazaki, 444-8585 Japan

**Keywords:** Oogenesis, Stem cells, Transcriptomics, Zoology

## Abstract

Many flatworms can alternate between asexual and sexual reproduction. This is a powerful reproductive strategy enabling them to benefit from the features of the two reproductive modes, namely, rapid multiplication and genetic shuffling. The two reproductive modes are enabled by the presence of pluripotent adult stem cells (neoblasts), by generating any type of tissue in the asexual mode, and producing and maintaining germ cells in the sexual mode. In the current study, RNA sequencing (RNA-seq) was used to compare the transcriptomes of two phenotypes of the planarian *Dugesia ryukyuensis*: an asexual OH strain and an experimentally sexualized OH strain. Pathway enrichment analysis revealed striking differences in amino acid metabolism in the two worm types. Further, the analysis identified serotonin as a new bioactive substance that induced the planarian ovary *de novo* in a postembryonic manner. These findings suggest that different metabolic states and physiological conditions evoked by sex-inducing substances likely modulate stem cell behavior, depending on their different function in the asexual and sexual reproductive modes. The combination of RNA-seq and a feeding assay in *D. ryukyuensis* is a powerful tool for studying the alternation of reproductive modes, disentangling the relationship between gene expression and chemical signaling molecules.

## Introduction

The alternation between asexual and sexual reproduction is a powerful reproductive strategy that allows organisms to benefit from both reproductive modes depending on circumstances. During asexual reproduction, a single individual is able to efficiently produce offspring without the need for mating partners, for example *via* budding, fragmentation, and fission; however, the offspring are genetically identical, *i.e*., lack genetic diversity. During sexual reproduction, two individuals genetically contribute to the offspring, increasing genetic variation, which gives the offspring a better chance to adapt to the changing environment. Quite a few organisms, especially members of the phylum Platyhelminthes, are able to employ both asexual and sexual reproduction modes^1^. Such a strategy appears to be beneficial to their reproductive success, as they are rapidly multiplying whilst retaining the genetic shuffling enabled by sex^2^. One of the most striking examples is parasitic flatworms, such as tapeworms and flukes, many of which have multiple hosts and complex life cycles, and employ asexual multiplication in an intermediate host and sexual reproduction in the definitive host. A recent study revealed that the numerical expansion in trematodes resulting from asexual multiplication within the intermediate host compensates for the poor transmission between the different hosts^3^.

A key to understanding the mechanism that underpins such a powerful reproductive strategy in Platyhelminthes is the control of pluripotent stem cells in different reproductive modes in these organisms. Platyhelminthes are recognized for their excellent ability to regenerate that is associated with their pluripotent stem cells known as neoblasts^4,5^. Further, there appears to be a clear link between asexual reproduction and regeneration capacity^6^. Although forms of asexual reproduction vary (*e.g*., architomy and paratomy, which are characterized by fission, before or after the formation of new organs, respectively), it is likely that the presence of pluripotent neoblasts that can generate any type of tissue represents a key feature in asexual reproduction, resulting in the multiple evolution of asexual reproduction as seen at least in basal Platyhelminthes^7^. Although little is known about the mechanisms underlying asexual reproduction, bioactive substance(s) from one species appear to facilitate asexual reproduction in other species^8^. Moreover, bioactive substances that induce the differentiation of neoblasts into germ cells and sexual reproduction (hereafter called “sex-inducing substances”) also exist^9–11^, and are broadly conserved beyond the species barrier, at least within the order Tricladida^12^. Collectively, these examples indicate the presence of a common molecular mechanism shared among flatworms, which controls the behavior of neoblasts depending on the reproductive mode; namely, switching between differentiation into various somatic tissues of a new clonal individual(s) in the asexual mode, and differentiation into germ cells in the sexual mode, in addition to the general stem cell functions, such as growth and tissue homeostasis^5,13^.

**Figure 1:**
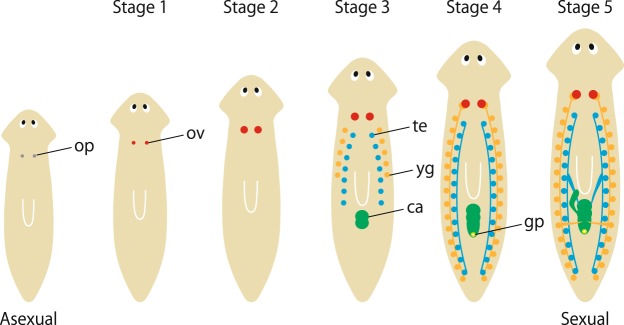
Five stages of sexualization in the planarian *D. ryukyuensis*. The worm begins to develop reproductive organs upon switching from the asexual to the sexual state. The process can be roughly divided into five stages based on the development of reproductive organs^14^. Briefly, the asexual worm possesses the ovarian primordia (op); the ovary (ov), with an increasing number of oogonia, starts to form during stage 1; the maturing ovaries with developing oocytes form during stage 2; the primordial testis (te), primordial yolk glands (yg)^35^, and copulatory apparatus (ca) form during stage 3; the genital pore (gp) becomes externally apparent during stage 4; the worm becomes sexually mature, ready for mating, and egg laying during stage 5. Note that the planarian body size changes because of the feeding procedure used for sexualization^10^: if the food does not contain sex-inducing substances, asexual worms become bigger without gonad development.

The planarian *Dugesia ryukyuensis* (phylum Platyhelminthes, class Turbellaria, order Tricladida) is an excellent model organism for studying the alternation between the asexual and sexual reproductive modes, for the following three reasons. First, the experimental system of inducing sexual reproduction in asexual worms is already established in *D. ryukyuensis*^10,14^ (Fig. 1). The OH strain of *D. ryukyuensis* is exclusively asexual, reproducing by transverse fission and subsequent regeneration; however, the worm can be experimentally sexualized by feeding it *Bdellocephala brunnea*^10,14^. This contrasts with another well-studied planarian, *Schmidtea mediterranea*, the reproductive mode of which appears to be impossible to switch because of a chromosomal translocation^15,16^. Experimentally controlled sexualization of the clonal OH strain provides a great opportunity for studying different gene expression patterns associated with the alternation between asexual and sexual phenotypes in the same genomic background, *i.e*., the manner in which sexuality is suppressed in an asexual individual and *vice versa*. Second, in general, the control of stem cells (*e.g*., self-renewal and differentiation) is inextricably linked to the dynamic physiological environment^17^. Although studies of other organisms indicate that stem cell behavior is strongly affected by nutrient availability^18,19^, *D. ryukyuensis* possesses an intriguing mechanism influencing stem cell behavior which is separate from its diet. Namely, the worm produces sex-inducing substances to maintain sexuality^10^, which likely affects the physiological status of the whole body. Hence, studying *D. ryukyuensis* may provide new insights into modulators of stem cell behavior. Third, the Tricladida order that *D. ryukyuensis* belongs to occupies an interesting position in the phylogeny of Platyhelminthes; it is closely related to the parasitic flatworm group, clade Neodermata, which consists of Monogenea, Cestoda (tapeworms), and Trematoda (flukes)^20^. Similarly to *Dugesia*, parasitic flatworms combine asexual and sexual reproduction at different phases of their complex life cycle, and their successful proliferation results in devastating parasitic diseases^21^. Growing evidence for the similarity of molecular signatures suggests that the complex life cycle of parasitic flatworms has evolved by adapting a developmental program already present in their free-living ancestors^22–24^. Thus, the study of *D. ryukyuensis* may provide useful insights for understanding their parasitic relatives.

The aim of the current study was twofold. First, we aimed to produce transcriptome catalogues of asexual and sexual *D. ryukyuensis* using RNA sequencing (RNA-seq). Second, we explored factors that contribute to the phenotypic and transcriptional plasticity of the reproductive modes in planarians, by focusing on asexual and experimentally sexualized worms of the OH strain.

## Results

### Development of Transcriptome Catalogues of Asexual and Sexual Planarians Using RNA-Seq

To characterize the *D. ryukyuensis* transcriptome and to examine differences in gene expression associated with the asexual and sexual modes of reproduction, we performed RNA-seq of the asexual, experimentally-induced sexual, and innate sexual planarians (see the Methods section; five biological replicates for each worm type). The innate sexual worms are sexual offspring of the OH strain, which become sexual without experimental manipulation^25^, and were included in the analysis to enrich the RNA-seq catalogue. On average, each sequencing library produced 15.2 M ± 6.6 M reads (mean ± SD) after quality-control filtering, yielding 228,246,618 reads in total (Supplementary Table S1). *De novo* assembly of transcript models was performed using all reads (Supplementary Dataset 1), and 181,393 contigs corresponding to 132,884 unigenes were identified (Supplementary Table S1). Moreover, 57,762 coding DNA sequences (CDSs) were predicted, and 51.5% of these (29,734 CDSs) were annotated (Supplementary Table S1). The reads were mapped to the transcript models with a mapping rate of >96% for all libraries (Supplementary Table S1).

To describe differences between the asexual and sexual modes of reproduction, an analysis of differentially expressed genes (DEG) in the RNA-seq data from asexual and experimentally-induced sexual (hereafter referred to as simply “sexual”) worms was performed. The innate sexual worms were excluded hereafter, because the present study focuses on the phenotypic plasticity of the OH strain with the same genetic background. In total, 11,795 DEGs were identified [likelihood ratio test, false discovery rate (FDR) < 0.01] (indicated in red in Supplementary Fig. S1), of which 1,191 were highly expressed in asexual worms [794 DEGs at a fold-change (FC) cutoff of >2] and 10,604 were highly expressed in the sexual worm (10,059 DEGs at FC cutoff of >2). These findings suggested that the majority of DEGs were biased towards sexual worms.

For an overview of the biological functions of the identified DEGs, we next performed a gene ontology (GO) term enrichment analysis (Supplementary Table S2). For the asexual DEGs, 8 out of the 27 enriched GO terms were linked to neurological processes, such as “transmission of nerve impulse”, “synaptic transmission”, “neurological system process”, “regulation of neurotransmitter levels”, “neuron differentiation”, “neuron projection development”, “neuron development”, and “neuromuscular synaptic transmission” (Supplementary Table S2). For the sexual DEGs, 154 GO terms were significantly enriched, 18 of which were involved in reproductive processes, consistent with the sexual phenotype, such as “DNA recombination”, “reproductive process in a multicellular organism”, “multicellular organism reproduction”, “gamete generation”, “male gamete generation”, “spermatogenesis”, “sexual reproduction”, “M phase of meiotic cell cycle”, “meiosis”, “meiotic cell cycle”, “male meiosis”, “meiosis I”, “reproductive structure development”, “gonad development”, “development of primary sexual characteristics”, “sex differentiation”, “reproductive developmental process”, and “reciprocal meiotic recombination” (Supplementary Table S2).

**Figure 2:**
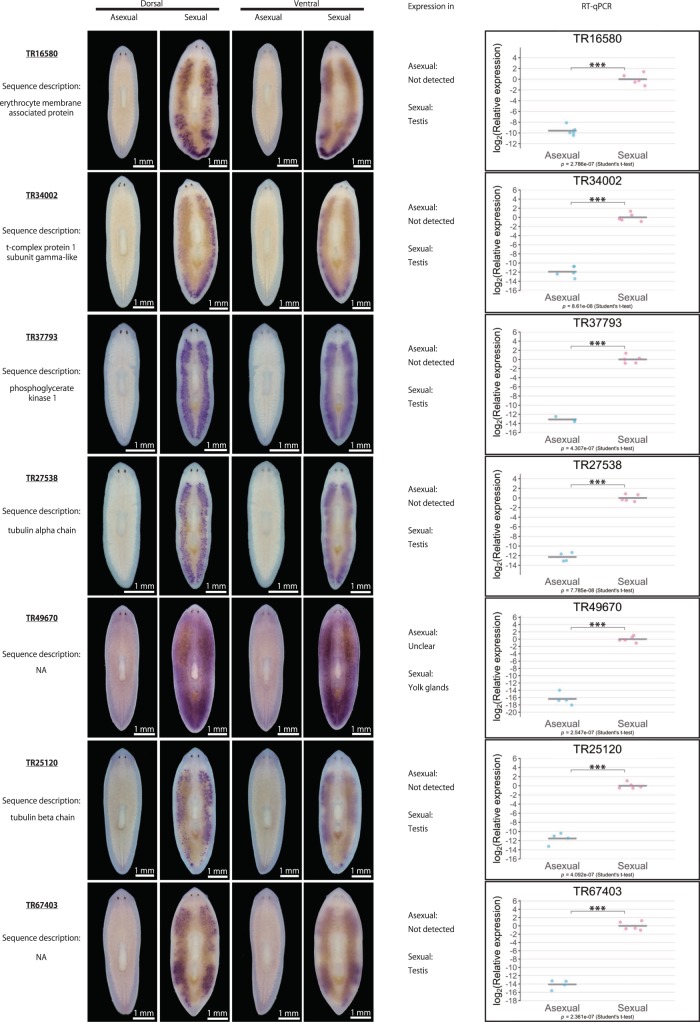

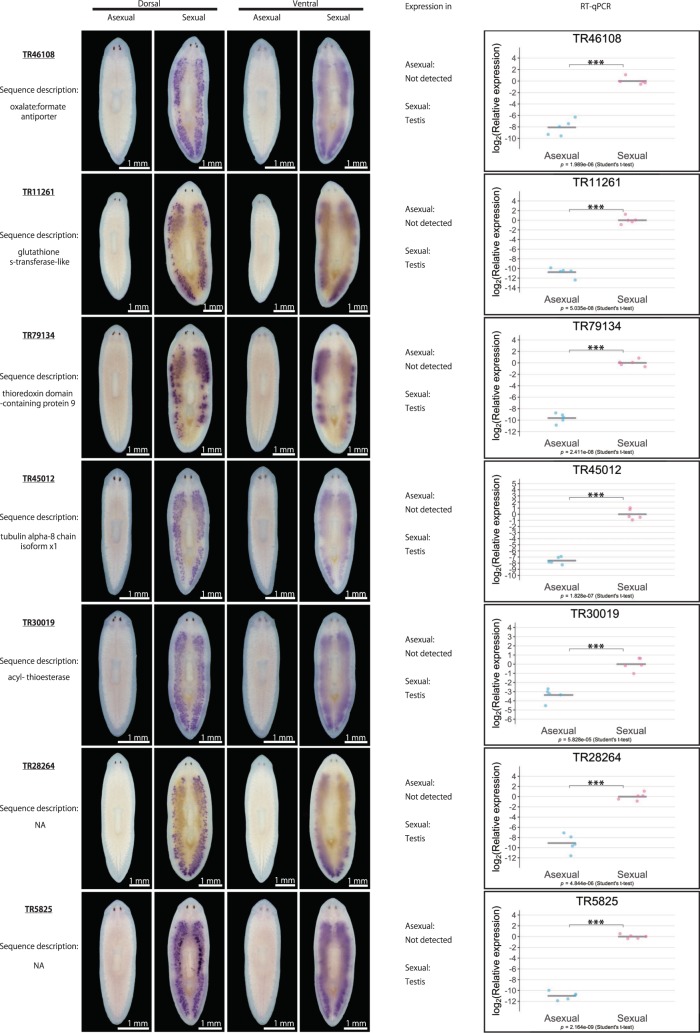

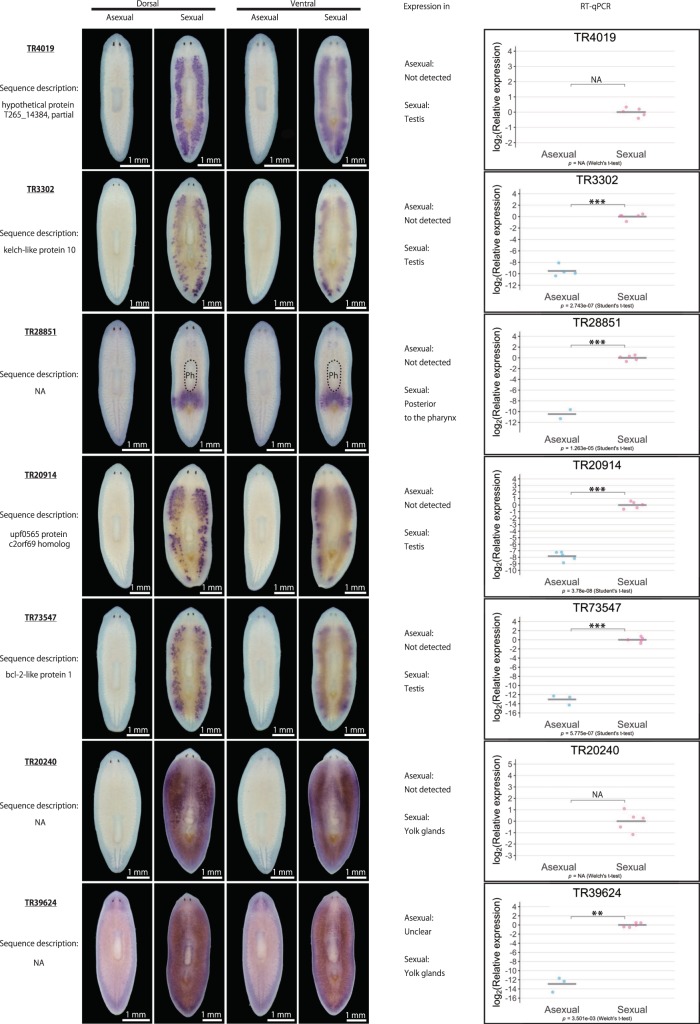
Qualitative and quantitative validation of sexual DEGs. Representative whole-mount *in situ* hybridization patterns for the ventral and dorsal sides of worms are shown. The expression pattern was judged based on three replicates. Signals were seen as blue/purple staining. The qRT-PCR data are shown relative to the expression level in the sexual worm, and log_2_ (relative expression) on the vertical axis indicates -ΔΔCt. Each circle indicates an individual asexual or sexual worm. Five replicates were used, but data are not shown if the expression was too low to be detected (handled as NA). The bars in the plots indicate the averages of -ΔΔCt. Asterisks indicate significant differences between the asexual and sexual worms (Student’s or Welch’s *t*-test: *P < 0.05; **P < 0.01; ***P < 0.001; n.s., not significant). Ph, pharynx.

We then examined the asexual and sexual DEGs in more detail. Table 1 lists the top 40 asexual and sexual DEGs, respectively, with the largest log_2_FC in terms of expression levels between the different modes of reproduction. From the top 25 asexual DEGs and top 40 sexual DEGs, we were able to design primers for 28 DEGs (seven DEGs for asexual worms and 21 DEGs for sexual worms; indicated in pink in Supplementary Fig. S1) to quantitatively verify their expression by quantitative reverse-transcription polymerase chain reaction (qRT-PCR) and qualitatively verify their expression by whole-mount *in situ* hybridization. For the seven asexual DEGs, whole-mount *in situ* hybridization revealed that four DEGs were expressed in the nervous system, including the brain and a pair of ventral nerve cords, while three of them were ubiquitously expressed (Supplementary Fig. S2). However, these expression patterns did not seem to be asexual specific, because they were also detected in sexual worms. For the remaining three asexual DEGs, TR46543|c5_g1_i5 was expressed in the ovarian primordium in the asexual worm, while the other two did not show distinct expression patterns. Moreover, qRT-PCR failed to confirm the asexual-biased expression of most of these seven asexual DEGs, with an exception of TR46543|c5_g1_i5, expression of which was higher in the asexual than in the sexual worms. On the other hand, qRT-PCR confirmed that the expression of all 21 sexual DEGs was significantly higher in sexual than in asexual worms (Fig. 2). Whole-mount *in situ* hybridization revealed that most of the sexual DEGs were expressed in the reproductive organs, such as the testes (17 DEGs) and yolk glands (3 DEGs). One exception was TR28851|c0_g1_i1, which was highly expressed in the region posterior to the pharynx (Fig. 2) in sexual worms. Overall, the main biological processes corresponding to these DEGs appeared to include neurological processes in asexual worms and reproductive processes in sexual worms, consistent with the trends of the GO enrichment analysis. However, these results suggest that the experimental validation of DEGs by whole-mount *in situ* hybridization and qRT-PCR, while reliable for sexual DEGs, was technically challenging for asexual DEGs. The results of the enrichment analysis for asexual DEGs should therefore be interpreted and handled with caution.

**Table 1:** Top 40 asexual and sexual DEGs. The DEGs are presented in descending order of log_2_FC; log_2_FC (asexual/sexual) for the asexual DEGs, and log_2_FC (sexual/asexual) for the sexual DEGs. Bold font indicates DEGs chosen for experimental validation by whole-mount *in situ* hybridization and qRT-PCR (DEG identification criteria: likelihood ratio test, FDR < 0.01).

Sexuality	ID	Length	logFC	logCPM	Likelihood ratio	*P-*value	FDR	Sequence description
Asexual	**TR47548|c3_g2_i4**	1736	7.77E + 00	1.12E + 00	6.77E + 01	1.88E - 16	8.77E - 15	NA
	TR47241|c4_g2_i8	439	7.38E + 00	−2.18E - 01	3.04E + 01	3.45E - 08	9.94E - 07	NA
	TR46139|c4_g2_i1	666	7.17E + 00	−3.83E - 01	1.63E + 01	5.42E - 05	1.05E - 03	NA
	**TR46930|c1_g1_i7**	1857	6.94E + 00	6.87E - 01	3.47E + 01	3.88E - 09	1.21E - 07	NA
	**TR46247|c1_g2_i3**	1025	6.94E + 00	−1.13E - 01	3.99E + 01	2.67E - 10	9.05E - 09	NA
	**TR46543|c5_g1_i5**	1621	6.48E + 00	−3.47E - 01	3.71E + 01	1.11E - 09	3.60E - 08	NA
	TR38806|c0_g1_i14	1721	6.40E + 00	−8.75E - 01	1.33E + 01	2.59E - 04	4.38E - 03	NA
	TR30470|c0_g3_i1	248	6.38E + 00	−9.94E - 01	1.75E + 01	2.80E - 05	5.68E - 04	NA
	**TR43664|c0_g1_i2**	1259	6.36E + 00	3.92E - 01	2.59E + 01	3.66E - 07	9.55E - 06	NA
	TR3030|c0_g1_i1	718	6.32E + 00	−1.07E + 00	2.12E + 01	4.18E - 06	9.54E - 05	ubiquitin-conjugating enzyme e2 k
	TR42765|c0_g1_i1	959	6.30E + 00	−8.67E - 01	1.31E + 01	3.03E - 04	5.05E - 03	NA
	TR46277|c2_g2_i1	533	6.26E + 00	1.91E - 01	2.24E + 01	2.18E - 06	5.16E - 05	tnf receptor-associated factor 6
	TR44416|c0_g2_i1	331	6.22E + 00	−1.20E + 00	2.53E + 01	4.83E - 07	1.24E - 05	NA
	TR46530|c0_g1_i3	1531	6.20E + 00	−9.00E - 01	1.21E + 01	5.00E - 04	7.91E - 03	NA
	TR47833|c9_g1_i1	227	6.18E + 00	−1.24E + 00	1.58E + 01	7.11E - 05	1.35E - 03	NA
	TR46881|c0_g1_i3	1303	6.11E + 00	−1.28E + 00	1.47E + 01	1.28E - 04	2.30E - 03	NA
	TR32727|c0_g2_i1	649	6.09E + 00	−1.27E + 00	1.93E + 01	1.13E - 05	2.43E - 04	NA
	**TR30587|c0_g1_i4**	853	6.03E + 00	−1.31E - 01	3.53E + 01	2.76E - 09	8.68E - 08	NA
	TR16992|c0_g1_i1	573	5.88E + 00	−1.37E + 00	1.71E + 01	3.57E - 05	7.10E - 04	NA
	**TR49031|c3_g1_i2**	3272	5.82E + 00	4.80E - 01	5.02E + 01	1.36E - 12	5.28E - 11	ras-related protein m-ras-like
	TR12695|c0_g1_i1	265	5.73E + 00	−1.46E + 00	1.39E + 01	1.92E - 04	3.35E - 03	NA
	TR38469|c1_g1_i1	599	5.73E + 00	−1.44E + 00	1.34E + 01	2.52E - 04	4.27E - 03	NA
	TR45705|c0_g1_i6	671	5.71E + 00	−1.41E + 00	1.18E + 01	5.84E - 04	9.10E - 03	NA
	TR46364|c2_g10_i3	707	5.71E + 00	−6.67E - 01	1.53E + 01	9.02E - 05	1.67E - 03	NA
	TR39276|c0_g2_i1	607	5.69E + 00	−1.39E + 00	1.70E + 01	3.79E - 05	7.52E - 04	NA
	TR41905|c0_g1_i6	1664	5.65E + 00	−1.60E + 00	1.28E + 01	3.53E - 04	5.80E - 03	NA
	TR45986|c10_g2_i2	1597	5.65E + 00	−1.24E + 00	1.86E + 01	1.60E - 05	3.38E - 04	NA
	TR33991|c0_g4_i3	427	5.65E + 00	−7.46E - 01	1.17E + 01	6.28E - 04	9.69E - 03	NA
	TR49422|c3_g3_i1	241	5.62E + 00	−1.49E + 00	1.58E + 01	6.88E - 05	1.30E - 03	NA
	TR5159|c0_g1_i1	1479	5.62E + 00	−4.16E - 01	2.92E + 01	6.52E - 08	1.83E - 06	NA
	TR47359|c4_g3_i2	667	5.59E + 00	−1.51E + 00	1.53E + 01	9.30E - 05	1.72E - 03	NA
	TR35175|c0_g2_i1	644	5.59E + 00	−6.89E - 01	1.74E + 01	3.09E - 05	6.22E - 04	NA
	TR49328|c1_g5_i2	2038	5.54E + 00	−1.32E + 00	1.48E + 01	1.20E - 04	2.17E - 03	histone-lysine n-methyltransferase setmar-like
	TR46269|c0_g3_i1	308	5.54E + 00	−1.48E + 00	1.95E + 01	1.02E - 05	2.20E - 04	NA
	TR41950|c1_g1_i2	609	5.50E + 00	−1.42E + 00	1.53E + 01	9.39E - 05	1.74E - 03	NA
	TR45263|c0_g1_i3	2117	5.39E + 00	−4.90E - 01	2.85E + 01	9.52E - 08	2.64E - 06	NA
	TR49456|c5_g1_i2	365	5.38E + 00	−1.79E + 00	1.51E + 01	1.01E - 04	1.85E - 03	NA
	TR48894|c0_g6_i1	235	5.37E + 00	−1.79E + 00	1.17E + 01	6.17E - 04	9.53E - 03	NA
	TR48635|c1_g2_i5	268	5.31E + 00	−1.84E + 00	1.20E + 01	5.34E - 04	8.39E - 03	hypothetical protein TcasGA2_TC012988
	TR48833|c5_g2_i3	256	5.31E + 00	−1.68E + 00	1.23E + 01	4.65E - 04	7.41E - 03	peptidoglycan recognition protein-1
Sexual	TR45015|c11_g2_i1	389	1.74E + 01	9.47E + 00	3.36E + 02	4.38E - 75	7.26E - 73	NA
	TR39624|c0_g1_i5	416	1.58E + 01	7.93E + 00	3.30E + 02	1.02E - 73	1.65E - 71	NA
	**TR16580|c0_g1_i1**	1604	1.52E + 01	7.27E + 00	8.10E + 02	3.47E - 178	2.33E - 174	erythrocyte membrane associated protein
	TR79276|c0_g1_i1	1255	1.48E + 01	6.88E + 00	8.48E + 02	1.87E - 186	2.61E - 182	sjchgc04698 protein
	**TR34002|c0_g1_i1**	1916	1.47E + 01	6.77E + 00	7.97E + 02	2.87E - 175	1.62E - 171	t-complex protein 1 subunit gamma-like
	**TR37793|c0_g1_i1**	2089	1.47E + 01	6.74E + 00	8.51E + 02	3.44E - 187	5.68E - 183	phosphoglycerate kinase 1
	**TR27538|c0_g1_i1**	2081	1.46E + 01	6.63E + 00	8.07E + 02	1.44E - 177	9.03E - 174	tubulin alpha chain
	**TR49670|c1_g1_i1**	347	1.45E + 01	8.10E + 00	3.72E + 02	6.74E - 83	1.27E - 80	NA
	TR30631|c1_g2_i1	376	1.45E + 01	6.54E + 00	6.76E + 02	6.13E - 149	5.82E - 146	NA
	**TR25120|c0_g1_i1**	1798	1.44E + 01	6.45E + 00	7.55E + 02	3.23E - 166	8.36E - 163	tubulin beta chain
	TR73196|c0_g1_i1	515	1.43E + 01	6.37E + 00	7.18E + 02	4.13E - 158	6.41E - 155	NA
	TR39208|c0_g1_i2	428	1.43E + 01	6.34E + 00	4.83E + 02	4.92E - 107	1.49E - 104	NA
	**TR67403|c0_g1_i1**	627	1.43E + 01	6.33E + 00	7.76E + 02	7.21E - 171	2.78E - 167	NA
	TR45486|c6_g3_i4	1107	1.42E + 01	7.85E + 00	9.12E + 02	2.16E - 200	7.85E - 196	tubulin beta-4b partial
	TR73406|c0_g1_i1	881	1.41E + 01	6.13E + 00	8.31E + 02	9.67E - 183	9.23E - 179	hypothetical protein
	TR18645|c0_g1_i1	1341	1.41E + 01	6.09E + 00	7.83E + 02	2.43E - 172	1.10E - 168	NA
	TR39624|c0_g1_i3	2365	1.40E + 01	1.06E + 01	2.90E + 02	5.91E - 65	8.35E - 63	NA
	**TR46108|c1_g3_i2**	1639	1.40E + 01	6.03E + 00	7.36E + 02	3.95E - 162	7.46E - 159	oxalate:formate antiporter
	**TR11261|c0_g1_i1**	728	1.40E + 01	6.01E + 00	7.10E + 02	1.78E - 156	2.43E - 153	glutathione s-transferase-like
	**TR79134|c0_g1_i1**	814	1.39E + 01	5.95E + 00	7.09E + 02	3.59E - 156	4.86E - 153	thioredoxin domain-containing protein 9
	TR55853|c0_g1_i1	1079	1.39E + 01	5.94E + 00	6.91E + 02	2.17E - 152	2.43E - 149	NA
	TR25043|c0_g1_i1	1156	1.39E + 01	5.93E + 00	7.07E + 02	9.55E - 156	1.28E - 152	NA
	TR33142|c0_g1_i1	885	1.39E + 01	5.92E + 00	6.77E + 02	2.45E - 149	2.39E - 146	expressed conserved protein
	TR18521|c0_g1_i2	272	1.39E + 01	5.91E + 00	7.15E + 02	1.75E - 157	2.52E - 154	NA
	TR29388|c0_g2_i1	447	1.38E + 01	5.88E + 00	4.07E + 02	2.03E - 90	4.44E - 88	NA
	**TR45012|c0_g1_i2**	1831	1.38E + 01	5.82E + 00	6.15E + 02	1.07E - 135	6.36E - 133	tubulin alpha-8 chain isoform x1
Sexual	**TR30019|c0_g1_i2**	1199	1.37E + 01	5.76E + 00	6.99E + 02	5.28E - 154	6.42E - 151	acyl- thioesterase
	**TR28264|c0_g1_i1**	3229	1.37E + 01	5.73E + 00	7.63E + 02	7.34E - 168	1.99E - 164	NA
	TR29267|c1_g1_i1	680	1.37E + 01	5.70E + 00	6.18E + 02	1.74E - 136	1.06E - 133	NA
	**TR5825|c0_g1_i1**	668	1.36E + 01	5.67E + 00	6.81E + 02	3.16E - 150	3.27E - 147	NA
	**TR4019|c0_g1_i1**	1694	1.36E + 01	5.66E + 00	6.26E + 02	3.47E - 138	2.26E - 135	hypothetical protein T265_14384, partial
	**TR3302|c0_g1_i1**	2422	1.36E + 01	5.66E + 00	7.67E + 02	7.74E - 169	2.26E - 165	kelch-like protein 10
	**TR28851|c0_g1_i1**	888	1.36E + 01	5.65E + 00	8.30E + 02	1.36E - 182	1.17E - 178	NA
	**TR20914|c0_g1_i1**	1281	1.36E + 01	5.63E + 00	6.10E + 02	1.23E - 134	7.17E - 132	upf0565 protein c2orf69 homolog
	**TR73547|c0_g1_i1**	822	1.36E + 01	5.62E + 00	5.97E + 02	9.13E - 132	4.92E - 129	bcl-2-like protein 1
	TR3479|c0_g1_i1	1295	1.35E + 01	5.56E + 00	6.78E + 02	1.89E - 149	1.86E - 146	cysteine and histidine-rich protein 1
	TR31160|c0_g1_i1	516	1.35E + 01	1.09E + 01	2.94E + 02	8.19E - 66	1.18E - 63	NA
	**TR20240|c0_g1_i1**	386	1.35E + 01	8.47E + 00	4.35E + 02	1.24E - 96	3.10E - 94	NA
	**TR39624|c0_g1_i1**	661	1.35E + 01	9.03E + 00	2.40E + 02	3.73E - 54	4.34E - 52	NA
	TR38673|c1_g1_i1	1708	1.35E + 01	8.76E + 00	5.90E + 02	2.60E - 130	1.33E - 127	NA

### Kyoto Encyclopedia of Genes and Genomes (KEGG) Pathway Enrichment Analysis Reveals Differences in Amino Acid Metabolism in Asexual and Sexual Worms

One of the most striking differences between asexual and sexual worms is the ability to self-produce sex-inducing substances to maintain sexuality^10^. This would be expected to drastically alter the worm’s physiological status and affect the regulation of neoblasts and/or germ cells. To identify the metabolic pathways contributing to the differences in these reproductive modes, we first performed KEGG^26–28^ pathway enrichment analysis for asexual and sexual DEGs and then validated some of the pathways of interest by verifying the reliability of the DEGs in these pathways by qRT-PCR.

**Figure 3:**
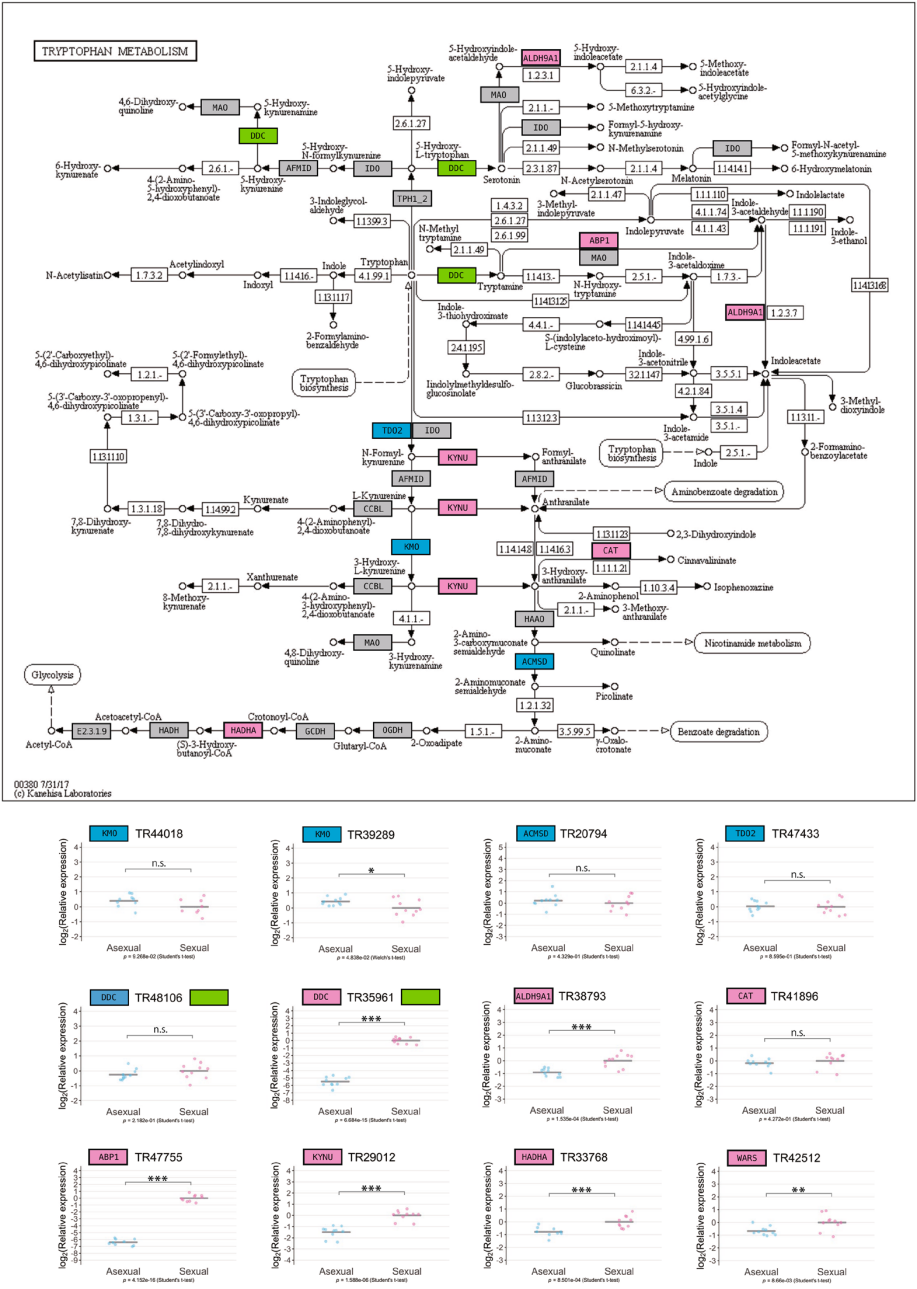
KEGG pathway mapping of the tryptophan metabolism. Annotated genes in RNA-seq analysis were mapped against KEGG pathway maps (www.kegg.jp/kegg/kegg1.html)^26–28^ using a KEGG mapper tool (http://www.kegg.jp/kegg/tool/map_pathway2.html). Genes identified as asexual DEGs are indicated in cyan, and genes identified as sexual DEGs are indicated in pink. A gene with three different isoforms that were identified as both asexual and sexual DEGs is indicated in green; specifically, TR48106|c0_g2_i2 is an asexual DEG; and TR35961|c1_g1_i1 and TR35961|c1_g1_i2 are sexual DEGs. Note that TR35961|c1_g1_i1 and TR35961|c1_g1_i2 were not distinguishable by qRT-PCR because the difference in length is only 33 bp, and thus these are shown as one gene. Genes not identified as DEGs but expressed in the planarian transcriptome in the present study are indicated in gray. The qRT-PCR data for each DEG are shown relative to the expression level in the sexual worm, and log_2_ (relative expression) on the vertical axis indicates -ΔΔCt. Each circle indicates an individual asexual or sexual worm. Eight to ten replicates were used, but data are not shown if the expression was too low to be detected or in the case of outliers (handled as NA). The bars in the plots indicate the averages of -ΔΔCt. Asterisks indicate significant differences between the asexual and sexual worms (Student’s or Welch’s *t*-test: *P < 0.05; **P < 0.01; ***P < 0.001; n.s., not significant). Note that TR42512, which encodes tryptophanyl-tRNA synthase, is not shown on the present map because it was mapped to the KEGG pathway of “aminoacyl-tRNA biosynthesis”, but the results of qRT-PCR are shown on the bottom right.

In total, 19 and 62 pathways were found to be potentially enriched in asexual and sexual worms, respectively, and some pathways were linked to amino acid metabolism (7 out of 19 pathways for asexual worms and 7 out of 62 pathways for sexual worms) (Table 2, Supplementary Table S3). Notably, tryptophan metabolism (Fig. 3); glycine, serine, and threonine metabolism (Supplementary Fig. S3); arginine and proline metabolism (Supplementary Fig. S4); and phenylalanine metabolism were enriched both in asexual and sexual worms, with different sets of genes contributing to the enrichment score of each pathway (Table 3). All 27 DEGs from the former three pathways were chosen for subsequent validation by qRT-PCR (Supplementary Table S4; also indicated in green in Supplementary Fig. S1). Three out of 14 asexual DEGs and 12 out of 13 sexual DEGs were confidently validated (Fig. 3 for DEGs in tryptophan metabolism; Supplementary Fig. S3 for glycine, serine, and threonine metabolism; and Supplementary Fig. S4 for arginine and proline metabolism), again revealing the difficulty in validating asexual DEGs. However, although only a few asexual DEGs were validated, our analysis clearly demonstrated that the same amino acid metabolic pathways are differentially regulated under different reproductive modes, with genes mainly up-regulated in the sexual state. These observations suggested that differences in these metabolic pathways may reflect a physiological state that suppresses or activates the production of endogenous sex-inducing substances.

**Table 2:** KEGG pathway enrichment analysis of asexual and sexual DEGs. Pathways indicated in bold represent amino acid metabolism pathways (DEG identification criteria: likelihood ratio test, FDR < 0.01).

Sexuality	Pathway	Fold enrichment	*P-*value	FDR
Asexual	**mmu00260:Glycine, serine and threonine metabolism**	237.1	4.18E - 11	3.13E - 09
	**mmu00350:Tyrosine metabolism**	334.4	1.07E - 09	4.02E - 08
	**mmu00380:Tryptophan metabolism**	204.6	9.37E - 07	2.34E - 05
	**mmu00330:Arginine and proline metabolism**	165.6	1.83E - 06	3.42E - 05
	mmu04080:Neuroactive ligand-receptor interaction	77.3	1.91E - 05	2.87E - 04
	mmu04810:Regulation of actin cytoskeleton	75.6	2.04E - 05	2.55E - 04
	mmu04510:Focal adhesion	75.6	2.04E - 05	2.55E - 04
	**mmu00360:Phenylalanine metabolism**	326.0	3.53E - 05	3.78E - 04
	mmu04514:Cell adhesion molecules (CAMs)	289.8	4.53E - 05	4.25E - 04
	mmu00620:Pyruvate metabolism	237.1	6.92E - 05	5.76E - 04
	**mmu00270:Cysteine and methionine metabolism**	200.6	9.80E - 05	7.35E - 04
	mmu05200:Pathways in cancer	43.5	1.08E - 04	7.33E - 04
	mmu05217:Basal cell carcinoma	173.9	1.32E - 04	8.23E - 04
	**mmu00250:Alanine, aspartate and glutamate metabolism**	163.0	1.50E - 04	8.67E - 04
	mmu00564:Glycerophospholipid metabolism	144.9	1.91E - 04	1.03E - 03
	mmu04666:Fc gamma R-mediated phagocytosis	124.2	2.62E - 04	1.31E - 03
	mmu04916:Melanogenesis	113.4	3.15E - 04	1.48E - 03
	mmu04270:Vascular smooth muscle contraction	93.1	4.70E - 04	2.07E - 03
	mmu04310:Wnt signaling pathway	84.1	5.76E - 04	2.40E - 03
Sexual	mmu04120:Ubiquitin mediated proteolysis	103.4	6.77E - 37	9.55E - 35
	mmu00230:Purine metabolism	94.5	3.49E - 29	2.46E - 27
	mmu04110:Cell cycle	122.4	6.71E - 28	3.16E - 26
	mmu05200:Pathways in cancer	63.6	2.62E - 27	9.23E - 26
	mmu03030:DNA replication	165.8	1.47E - 24	4.14E - 23
	mmu03430:Mismatch repair	226.6	1.16E - 22	2.73E - 21
	mmu04114:Oocyte meiosis	140.9	1.03E - 17	2.07E - 16
	mmu03440:Homologous recombination	172.2	9.45E - 17	2.00E - 15
	mmu04914:Progesterone-mediated oocyte maturation	153.0	3.00E - 14	4.70E - 13
	mmu03420:Nucleotide excision repair	92.7	4.74E - 14	6.68E - 13
	mmu00240:Pyrimidine metabolism	60.9	7.30E - 14	9.35E - 13
	**mmu00380:Tryptophan metabolism**	110.3	2.95E - 11	3.47E - 10
	mmu03018:RNA degradation	64.9	3.51E - 11	3.81E - 10
	mmu00052:Galactose metabolism	200.8	3.77E - 11	3.79E - 10
	mmu00970:Aminoacyl-tRNA biosynthesis	81.5	2.36E - 10	2.22E - 09
	mmu04612:Antigen processing and presentation	114.8	1.32E - 09	1.17E - 08
	mmu03040:Spliceosome	27.4	1.60E - 09	1.33E - 08
	mmu00520:Amino sugar and nucleotide sugar metabolism	107.1	1.98E - 09	1.55E - 08
	mmu04622:RIG-I-like receptor signaling pathway	95.6	1.81E - 07	1.34E - 06
	mmu03410:Base excision repair	89.3	2.46E - 07	1.73E - 06
	**mmu00330:Arginine and proline metabolism**	63.8	1.06E - 06	7.11E - 06
	mmu05211:Renal cell carcinoma	60.9	1.29E - 06	8.27E - 06
	mmu00510:N-Glycan biosynthesis	60.9	1.29E - 06	8.27E - 06
	mmu04144:Endocytosis	30.3	1.68E - 06	1.03E - 05
	**mmu00340:Histidine metabolism**	133.9	2.80E - 06	1.64E - 05
	mmu00500:Starch and sucrose metabolism	133.9	2.80E - 06	1.64E - 05
	mmu04910:Insulin signaling pathway	49.6	3.05E - 06	1.72E - 05
	mmu04310:Wnt signaling pathway	43.2	5.41E - 06	2.93E - 05
	mmu03050:Proteasome	39.4	7.90E - 06	4.12E - 05
	mmu00010:Glycolysis / Gluconeogenesis	97.4	8.18E - 06	4.12E - 05
	mmu04142:Lysosome	35.2	1.24E - 05	6.04E - 05
	mmu02010:ABC transporters	82.4	1.41E - 05	6.63E - 05
	mmu04540:Gap junction	51.0	6.42E - 05	2.92E - 04
	mmu05213:Endometrial cancer	48.7	7.41E - 05	3.26E - 04
	mmu04916:Melanogenesis	46.6	8.50E - 05	3.63E - 04
	mmu05210:Colorectal cancer	44.6	9.68E - 05	4.02E - 04
	mmu04912:GnRH signaling pathway	41.2	1.24E - 04	4.98E - 04
	mmu01040:Biosynthesis of unsaturated fatty acids	160.7	1.37E - 04	5.35E - 04
Sexual	mmu04062:Chemokine signaling pathway	36.9	1.72E - 04	6.57E - 04
	mmu00903:Limonene and pinene degradation	133.9	2.04E - 04	7.58E - 04
	mmu05340:Primary immunodeficiency	133.9	2.04E - 04	7.58E - 04
	mmu04020:Calcium signaling pathway	34.6	2.11E - 04	7.63E - 04
	mmu05016:Huntington’s disease	17.2	2.14E - 04	7.53E - 04
	**mmu00360:Phenylalanine metabolism**	100.4	3.80E - 04	1.30E - 03
	mmu04115:p53 signaling pathway	80.3	6.07E - 04	2.04E - 03
	mmu00983:Drug metabolism	80.3	6.07E - 04	2.04E - 03
	mmu04810:Regulation of actin cytoskeleton	23.3	6.84E - 04	2.24E - 03
	**mmu00410:beta-Alanine metabolism**	73.0	7.40E - 04	2.37E - 03
	mmu00620:Pyruvate metabolism	73.0	7.40E - 04	2.37E - 03
	mmu04630:Jak-STAT signaling pathway	66.9	8.86E - 04	2.77E - 03
	mmu00640:Propanoate metabolism	66.9	8.86E - 04	2.77E - 03
	mmu05212:Pancreatic cancer	61.8	1.04E - 03	3.20E - 03
	mmu05012:Parkinson’s disease	19.1	1.22E - 03	3.64E - 03
	mmu00480:Glutathione metabolism	53.6	1.40E - 03	4.10E - 03
	mmu05014:Amyotrophic lateral sclerosis (ALS)	50.2	1.60E - 03	4.58E - 03
	mmu05414:Dilated cardiomyopathy	42.3	2.26E - 03	6.35E - 03
	mmu03022:Basal transcription factors	42.3	2.26E - 03	6.35E - 03
	mmu03020:RNA polymerase	40.2	2.50E - 03	6.90E - 03
	mmu05222:Small cell lung cancer	40.2	2.50E - 03	6.90E - 03
	mmu04012:ErbB signaling pathway	36.5	3.03E - 03	8.18E - 03
	**mmu00260:Glycine, serine and threonine metabolism**	36.5	3.03E - 03	8.18E - 03
	**mmu00280:Valine, leucine and isoleucine degradation**	33.5	3.60E - 03	9.54E - 03

**Table 3:** DEGs in the KEGG pathways enriched both in asexual and sexual worms, with different sets of genes contributing to the enrichment score. Pathways indicated in bold are amino acid metabolism pathways (DEG identification criteria: likelihood ratio test, FDR < 0.01).

Pathway	Asexual		Sexual	
	Count	KEGG gene name	Count	KEGG gene name
**mmu00260:Glycine, serine and threonine metabolism**	6	CTH, DAO, DLD, DMGDH, PHGDH, TDH	3	AGXT2, GNMT, PSPH
**mmu00380:Tryptophan metabolism**	4	ACMSD, DDC, KMO, TDO2	7	ABP1, ALDH9A1, CAT, DDC, HADHA, KYNU, WARS
**mmu00330:Arginine and proline metabolism**	4	ACY1, CPS1, DAO, GOT1	5	ABP1, ACY1, ALDH9A1, AMD1, SRM
mmu04810:Regulation of actin cytoskeleton	4	GSN, ITGAV, MYLK, WAS	4	APC2, ENAH, IQGAP1, PXN
**mmu00360:Phenylalanine metabolism**	3	DDC, GOT1, HPD	3	DDC, PAH, PRDX6
mmu00620:Pyruvate metabolism	3	DLD, GRHPR, LDHD	3	ALDH9A1, MDH1, ME2
mmu05200:Pathways in cancer	4	AXIN2, FZD4, ITGAV, WNT4	19	APC2, ARNT, BCL2L1, BRCA2, CASP8, CBL, CCDC6, CDH1, CUL2, MLH1, MSH2, MSH6, PIAS1, RAD51, RBX1, SLC2A1, SUFU, TCEB1, TRAF3
mmu04916:Melanogenesis	3	FZD4, TYR, WNT4	4	ADCY1, ADCY2, ADCY5, TYR
mmu04310:Wnt signaling pathway	3	AXIN2, FZD4, WNT4	5	APC2, CACYBP, CSNK2B, RBX1, RUVBL1

Hereafter, we focus on the enriched pathways in the sexual state. Notably, the “insulin signaling pathway” was one of the enriched pathways in the sexual worm (FDR = 1.72E-05) (Tables 2 and 4). From the point of view of somatic and germline stem cell regulation by nutrient-sensing pathways^18,19^, another major nutrient-sensing pathway, the mechanistic target of rapamycin (mTOR) signaling pathway, was also searched for but was not identified among the enriched pathways. For most analyses, DEGs identified at an FDR < 0.01 were used, to focus on genes that were differentially expressed, and as unequivocally as possible, between asexual and sexual worms, and to narrow down the findings to specific, reliably enriched pathways. However, when DEGs were identified using more relaxed significance criteria, namely, an FDR < 0.05 instead of 0.01, “mTOR signaling pathway” was indeed identified by the pathway enrichment analysis (FDR = 4.89E-05) (Table 4).

**Table 4:** Insulin signaling and mTOR signaling pathways enriched when sexual DEGs were identified using strict (likelihood ratio test, FDR < 0.01) and relaxed (likelihood ratio test, FDR < 0.05) statistical criteria.

Criteria for DEG identification	Sexuality	Pathway	Count	KEGG gene name	Fold enrichment	*P-*value	FDR
FDR < 0.01	Sexual	mmu04910:Insulin signaling pathway	5	CBL, EIF4E, EIF4EBP1, ELK1, G6PC	49.6	3.05E-06	1.72E-05
FDR < 0.05	Sexual	mmu04910:Insulin signaling pathway	5	CBL, EIF4E, EIF4EBP1, ELK1, G6PC	41.1	6.40E-06	3.09E-05
	Sexual	mmu04150:mTOR signaling pathway	4	CAB39, EIF4B, EIF4E, EIF4EBP1	88.9	1.05E-05	4.89E-05

### Serotonin is One of the Ovary-Inducing Substances

It was previously reported that tryptophan is one of the sex-inducing substances that do not fully sexualize worms, but induce *de novo* ovaries^29^. Although the ovary-inducing activity of d-tryptophan is 500 times higher than that of l-tryptophan, the administration of a large amount of l-tryptophan (300 μg/worm/d) is also effective, suggesting that elevated levels of l-tryptophan–derived metabolites, including d-tryptophan, might affect ovary development^29^. Since tryptophan metabolism genes were enriched in sexual worms, we focused on a neurotransmitter, serotonin (5-hydroxytryptamine), which is a tryptophan metabolite. Serotonin is synthesized from 5-hydroxytryptophan by 3,4-dihydroxyphenylalanine decarboxylase (DDC). The RNA-seq analysis and subsequent validation by qRT-PCR revealed that *D. ryukyuensis* expresses DDC, encoded by sexual DEGs (TR35961|c1_g1_i1 and TR35961|c1_g1_i2, with a 33-bp difference in length, apparently encoding different isoforms of the enzyme) (Fig. 3). This suggested that serotonin synthesis may be differentially up-regulated in the sexual state.

**Figure 4:**
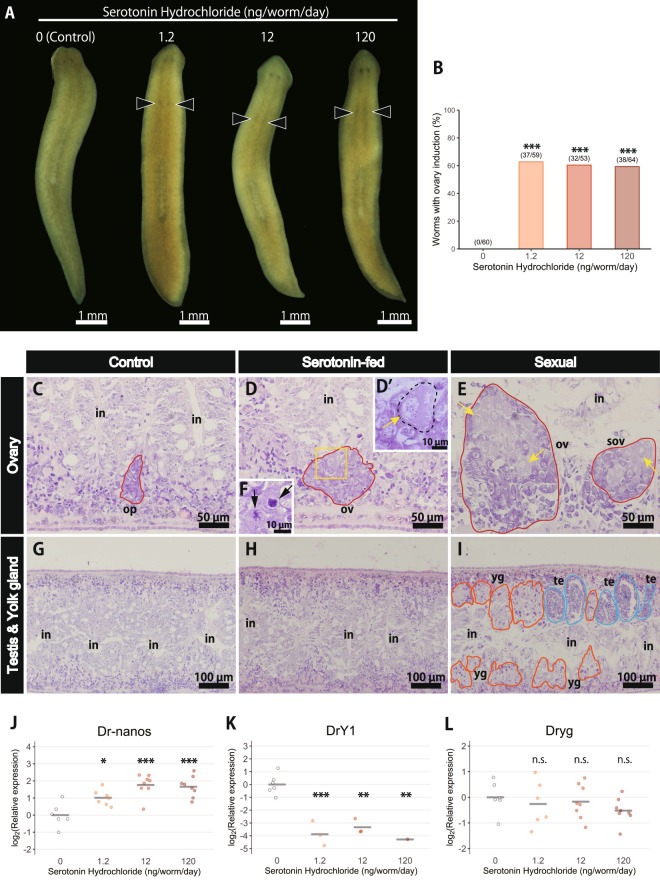
Serotonin is an ovary-inducing substance. (**A**) Images of the worms after 3 weeks of serotonin feeding. Induced ovaries (arrowhead) can be observed in the serotonin-fed groups but not in the control group. (**B**) Percentage of worms that developed externally observable ovaries (arrowhead in A) is shown (Fisher’s exact test: ***P < 0.001). Scoring was done at the completion of 4 weeks of serotonin feeding. Sample sizes were n = 60 for the control group; n = 59 for the group fed a low serotonin concentration (1.2 ng/worm/d); n = 53 for the group fed an intermediate serotonin concentration (12 ng/worm/d); and n = 64 for the group fed a high serotonin concentration (120 ng/worm/d). (**C**–**H**) The control, serotonin-fed, and experimentally sexualized worms were sagittally sectioned and stained with HE. The dorsal sides are at the top and the anterior sides are at the left. Only an ovarian primordium was found in the control worm (**C**), while ovaries were found in the serotonin-fed (**D**) and sexual worms (**E**). The cells indicated by the yellow arrow are an oocyte. The ovary of the serotonin-fed worm included an oocyte (D′, high magnification of the yellow box, dotted line) and a few dividing cells in a different part of that ovary (**F**, black arrow), as characterized by the nuclei with clumped chromatin darkly stained with HE. Neither the control (**G**) nor serotonin-fed (**H**) worms formed a testis or yolk gland, while these organs were found in the sexual worm (**I**). Domains bounded by the red line, the blue line, and the orange line are the female germ cell masses (ovaries), the male germ cell masses (testes), and yolk glands, respectively. op, ovarian primordium; ov, ovary; sov, supernumerary ovary; te, testis; yg, yolk gland; in, intestine. (**J**–**L**) The induction of other reproductive organs was examined by qRT-PCR analysis using the germ cell marker gene *Dr-nanos* (**J**), the testis marker gene *DrY1* (**K**), and the yolk gland marker gene *Dryg* (**L**). The qRT-PCR data for each DEG are shown relative to the expression level in the control worm, and log_2_ (relative expression) on the vertical axis indicates -ΔΔCt. Each circle indicates an individual worm in the control or serotonin-fed groups. Eight replicates were used, but data are not shown if the expression was too low to be detected or in the case of outliers (handled as NA). The bars in the plots indicate the averages of -ΔΔCt. Asterisks indicate significant differences between the asexual and sexual worms (Tukey’s HSD test: *P < 0.05; **P < 0.01; ***P < 0.001; n.s., not significant). Note that *DrY1* and *Dryg* were detected in the control (asexual) group due to the high sensitivity of qRT-PCR, but neither the testis nor the yolk glands were morphologically observed in the asexual worms.

Hypothesizing that serotonin may play an important role in the sexualization of planarians, a bioassay was conducted by feeding asexual worms serotonin. In fact, when the asexual worms were fed serotonin (hydrochloride salt) at 1.2, 12, or 120 ng/worm/d, the worms successfully developed ovaries (Fig. 4A). There were significantly more worms with such externally observed ovaries in the serotonin-fed groups than in the control group (Fig. 4B; Fisher’s exact test, p < 0.001 for all three concentrations). The copulatory apparatus was not observed in any groups (Fig. 4A).

As other reproductive organs, such as the testis and yolk glands, are not visible under a microscope, the formation of the reproductive organs was examined in more detail by histology and qRT-PCR. First, only an ovarian primordium was observed in the control worm (Fig. 4C); here, ovarian primordium is defined as a very small cell cluster in asexual worms that expresses the germ-cell marker gene *Dr-nanos*^30^, located in a region corresponding to the ovary region in sexual worms. Meanwhile, ovaries were found in the serotonin-fed (Fig. 4D) and sexual worms (Fig. 4E). In *D. ryukyuensis*, the ovary from stage 2 onwards (Fig. 1) consists of oogonia and oocytes, with oogonia located mainly in the periphery, and oocytes located more centrally in the ovary^10^. Moreover, oocytes are distinguished from oogonia by their larger size and a lower nucleus/cytoplasm ratio (Fig. 4E, yellow arrows). Notably, an oocyte was found in the ovary of the serotonin-fed worm (Fig. 4D′, yellow arrow), along with a few dividing cells in a different part of that ovary (Fig. 4F, black arrows), although it was impossible to distinguish between mitotic and meiotic divisions. These results suggest that 4 weeks of serotonin feeding induced a stage 2 ovary. Moreover, the serotonin-fed worm possessed supernumerary ovaries (Supplementary Fig. S5A, indicated as “sov”), which are extra ectopic ovaries in addition to the main ovaries, and are often observed in experimentally sexualized worms of *D. ryukyuensis* (Fig. 4E, indicated as “sov”; also see previous studies^25,31^). The formation of the testis and yolk gland was observed neither in the control (Fig. 4G) nor in the serotonin-fed (Fig. 4H) worms via histological analysis, while these organs were found in the sexual worm (Fig. 4I).

Next, qRT-PCR analysis was performed to evaluate the formation of reproductive organs, as histological analysis can be applied to only a limited number of worms. The expression level of the germ-cell marker gene *Dr-nanos*^30^ was significantly higher in the serotonin-fed groups than in the control group (Fig. 4J). Meanwhile, the testis marker gene *DrY1* (previously reported as DrC_00456^30,32^), a homolog of *DeY1* in *Dugesia etrusa*^33^ and *MSY4* in *S. mediterranea*^34^, was not detected in many worms in the serotonin-fed groups (4 out of 7 worms, 5 out of 8 worms, and 7 out of 8 worms fed a low, intermediate, and high concentration of serotonin, respectively), thus resulting in apparently significant differences between the control and serotonin-fed groups (Fig. 4K). While no ovary-specific marker for *D. ryukyuensis* is currently available, these results indirectly suggest that serotonin induced a *Dr-nanos*-expressing organ other than the testis, *i.e*. the ovary, consistent with the results of the histological analysis. Moreover, the yolk gland marker gene *Dryg*^35^ was not significantly up-regulated (Fig. 4L), suggesting that the serotonin-fed worms did not form yolk glands. Note that *DrY1* and *Dryg* were detected in the control (asexual) group, due to the high sensitivity of qRT-PCR, but neither the testis nor the yolk glands were morphologically observed in the asexual worms. The Ct values for *DrY1* and *Dryg* in the control group were also over 30 for almost all samples, indicating that the expression level was very low.

Serotonin is a neurotransmitter that may potentially influence planarian physiology, but the worms appeared normal and behaved normally throughout the experiment, at least within the range of serotonin concentrations used in this study. Body size was not significantly affected by serotonin feeding (Supplementary Fig. S5B). Moreover, fissioning was rarely observed in each group (Supplementary Fig. S5C). Thus, overall, serotonin induced the ovary, but not other reproductive organs such as the testis, yolk glands, and copulatory apparatus; these results demonstrated that serotonin is one of the ovary-inducing substances in *D. ryukyuensis*.

## Discussion

In the current study, we generated a catalogue of transcripts associated with the different reproduction modes of the planarian *D. ryukyuensis*. By investigating the expression pattern of DEGs with high fold expression changes between the asexual and sexual modes, we found that most sexual DEGs were expressed in the testis or yolk gland, which was reasonable given that these organs are numerous and distributed throughout the planarian body (Fig. 1).

The yolk gland is a unique reproductive organ in planarians; it produces yolk cells, which are packed together with several fertilized eggs, forming a polyembryonic egg capsule termed a cocoon (yolk cells vastly outnumber fertilized eggs in a cocoon)^36,37^. In addition to providing a nutritional support for the embryos, the yolk glands play a key role of producing and/or storing sex-inducing substances, as asexual worms can be fully sexualized when fed cocoons, regardless of whether the cocoons are laid by *D. ryukyuensis* (*i.e*., same species) or *B. brunnea* (*i.e*., different species)^12^. Moreover, sex-inducing substances seem to be conserved across species, and the efficacy of induction has been confirmed for gland-harboring flatworms (*i.e*., *B. brunnea* and the terrestrial flatworm *Bipalium nobile*) but not for species lacking yolk glands (*i.e*., the marine flatworm *Thysanozoon brocchii*)^12^. If the genes in the enriched pathways identified in the present study, especially pathways involving amino acid metabolism, were expressed in the yolk glands, they would be strong candidates responsible for the synthesis of sex-inducing substances. Moreover, none of the three sexual DEGs expressed in the yolk glands identified in the current study (Fig. 2) were annotated (Table 1), while most of the DEGs in the testis (14 out of 17 DEGs; Fig. 2) were annotated (Table 1), corroborating the findings of previous studies. For example, tubulin homologs (TR27538|c0_g1_i1, TR25120|c0_g1_i1, and TR45012|c0_g1_i2; Fig. 2) and a homolog of t-complex protein 1 subunit gamma (also known as CCT3) (TR34002|c0_g1_i1; Fig. 2) were also expressed in the testis of *S. mediterranea*^34,38^. This contrast could conceivably reflect the uniqueness of the flatworm yolk gland. The transcriptome catalogue generated in the present study will be useful for exploring not only annotated genes, but also novel non-annotated genes in the yolk glands, including non-coding RNAs and transcripts encoding signaling peptides. Given that the reproductive system with the distinct yolk cell and oocyte generating organs is shared among planarians and descending parasitic flatworms^20^, exploring the functions of yolk gland genes and phylogenetic comparisons may provide new insights into the flatworm reproductive strategy, namely, the evolutionary origin of sex-inducing substances, their conservation within flatworms, and their evolutionary and biological significance.

The expression of asexual DEGs was often detected both in asexual and sexual worms. For example, in the case of the asexual DEG TR47548|c3_g2_i4, which was detected in the brain, ovary, and testis by whole-mount *in situ* hybridization (Supplementary Fig. S2), eight possible isoforms were identified in the RNA-seq data, and five of these were found in the sexual DEG list. This may reflect a limitation of the technique in cases in which it is difficult to design specific probes for distinguishing among different isoforms, suggesting that searching for candidate genes responsible for asexuality using a gene expression pattern-based approach may be challenging. Other plausible explanations for the failure of experimental validation of many asexual DEGs is that we did not use a low-read cut-off during the identification of DEGs. This may have largely increased the number of false positives, including those which could not be validated by qRT-PCR. However, RNA-seq is useful in terms of obtaining a big picture of the gene network, as in the present study, it revealed the potential importance of amino acid metabolism. By combining this method with careful validation of the DEGs by subsequent qRT-PCR, this approach effectively narrowed down the number of potential candidate genes for future functional analysis involving gene silencing using RNA interference.

The most striking finding of the current study was that several amino acid metabolic pathways were enriched in, at least, sexual worms; specifically, tryptophan metabolism was enriched and differentially regulated, with one validated asexual DEG and 6 validated sexual DEGs. In a previous study, we showed that tryptophan plays an important role in planarian sexualization^29^. The amount of tryptophan in the sexual *D. ryukyuensis* was about 25 times higher than that in asexual worms, with a ratio of d-tryptophan to l-tryptophan of 0.014. In fact, l- and d-tryptophan were verified as the bioactive substances required for ovarian development in *D. ryukyuensis*; ovarian development is a key step, necessary for sexualization to proceed, and one that always precedes the development of other reproductive organs.

Interestingly, we also found that the glycine, serine, and threonine metabolism pathway and the arginine and proline metabolism pathway were differentially enriched in both reproductive modes, with d-amino acid oxidase (DAO), one of the experimentally validated asexual DEGs in the present study, participating in both pathways in asexual worms (Table 3 and Supplementary Figs S3 and S4). DAO degrades d-amino acids. In *D. ryukyuensis*, the ovary-inducing activity of d-tryptophan is 500 times higher than that of l-tryptophan^29^. The importance of other d-amino acids in planarians was previously reported^39^. The ovarian-inducing activity of four amino acids (d-arginine, d-phenylalanine, d-leucine, and d-asparagine) was confirmed in exogenous feeding experiments, with d-arginine exhibiting the highest bioactivity (higher than that of d-tryptophan). Although the presence of endogenous forms of these d-amino acids is yet to be confirmed, a *DAO* homolog in *D. ryukyuensis*, *Dr-DAO*, is expressed throughout the body in asexual worms to decrease the level of d-amino acids, hence suppressing the undesired ovarian development^39,40^. Consistently, the overall *Dr-DAO* expression gradually decreases with the progression of sexualization; however, the transient increase of *Dr-DAO* expression in the ovaries seems to be required for the establishment of functional ovaries^39,40^. Although further research is required to clarify the role of *Dr-DAO*, it is encouraging that the pathway enrichment analysis presented in the current study independently supported the importance of amino acid metabolism, including the balance between production and degradation of enantiomers, in the regulation of asexuality and sexuality.

In the current study, we identified serotonin as a new ovary-inducing substance. Compared with the administration of l-tryptophan (300 μg/worm/d) in a previous study^29^, lower amounts of serotonin (1.2 ng/worm/d) were effective, which corroborated the idea that serotonin is one of l-tryptophan–derived metabolites responsible for ovary induction, besides d-tryptophan. Growing evidence suggests the role of serotonin in the regulation of oocyte maturation in a wide variety of organisms. *E.g*., the following were observed: stimulatory effect of serotonin on the ovarian development in prawns^41^; selective expression of several serotonin receptors in an avian germ cell^42^; inhibitory effect of serotonin antagonists on oocyte maturation in starfish and a stimulatory effect in an amphibian^43^; and high levels of serotonin in the human ovarian follicular fluid, fluctuating with the ovulatory cycle^44^. In *D. ryukyuensis*, it is notable that serotonin induced the ovary *de novo* in an adult asexual worm in a postembryonic manner. The ovary induced by serotonin feeding was stage 2, with an oocyte and a few dividing cells (Fig. 4D′ and F, respectively). In the closely related species *Dugesia japonica*, ovarian and testicular primordia in asexual worms were also characterized by the expression of *Djnos*, a homolog of *nanos* in *D. japonica*^45^. Intriguingly, the labeling of proliferating cells by 5′-bromo-2′-deoxyuridine (BrdU) revealed that the cell cycle of these *Djnos*-positive cells seemed to be arrested in asexual worms^45^. If this is also the case in *D. ryukyuensis*, the possible functional role of serotonin in the present study may be to activate the arrested cell cycle of germline stem cells in ovarian primordia, promoting the proliferation and differentiation of female germ cells (*i.e*. oogonia and oocytes). Moreover, asexual worms generally have only one pair of ovarian primordia. Supernumerary ovaries observed in the serotonin-fed worm may also suggest an alternative functional role of serotonin in inducing the differentiation of neoblasts into female germline stem cells and/or oogonia, resulting in the establishment of extra ovaries outside of the pair of main ovaries. These two roles are not mutually exclusive but should be clearly disentangled in future studies.

Additionally, whether the observed ovary induction is regulated directly by serotonin and/or via a serotonergic neuron should be examined in more detail. Thus far, tryptophan hydroxylase (TPH), which is generally used as a serotonergic neuron marker, has not been identified as a DEG but is predicted to be involved in the tryptophan metabolism in *D. ryukyuensis* (Fig. 3). In the planarian *D. japonica*, TPH-positive neurons have been identified already, indicating the presence of serotonergic neurons^46^. In the future, examining the spatial distribution of TPH-positive cells and that of the serotonin receptor may aid in understanding the mechanism of germ cell differentiation mediated by serotonin. The present data indicate that the combination of RNA-seq and a feeding assay that enables the observation of *de novo* germ cell differentiation in the adult *D. ryukyuensis* will constitute a powerful tool for studying the molecular mechanisms underlying germ cell differentiation, disentangling the interaction between chemical signaling molecules and gene expression patterns. A detailed investigation of the differently enriched metabolic pathways may contribute to the identification of sex-inducing substances in the future.

Although experimental sexualization in the system used in this study was induced by feeding asexual worms sexual worms of other species, this unlikely affected the results of our pathway enrichment analysis, as the asexual and sexual worms were maintained on chicken livers for 1 year prior to the construction of the RNA-seq libraries, to exclude the effect of different diets. Therefore, the observed differences in the enriched pathways are likely due to the different reproduction modes; namely, the differential physiological conditions, impacted by the presence or absence of sex-inducing substances, and the differing control needs of the somatic and germline stem cells as per different reproductive modes.

Similar approaches were used in another planarian, *S. mediterranea*, to study the genetic basis of the hermaphroditic reproductive system using microarrays and transcriptomic analysis. Many DEGs in the reproductive system were successfully identified by such studies, including transcription factors, RNA-interacting genes, and genes involved in signal transduction^34,47,48^. In a study comparing the asexual and sexual strains of this species by microarray^47^, Cluster of Orthologous Group (COG) analyses also identified a difference in “amino acid transport and metabolism” (4.9% of sexually up-regulated genes with putative roles, as shown in the supplementary file), although this difference was not much discussed. Compared to regulatory genes expressed in reproductive organs, differences in such metabolic pathways may be difficult to attribute to differences in reproductive modes, because the possibility that the differences simply stem from general differences between strains with different genetic backgrounds, rather than from differences in the reproductive modes of interest, cannot be ruled out. The present study using *D. ryukyuensis* clearly demonstrated that the identified differences in amino acid metabolism are epigenetically induced in association with the switch from asexual to sexual mode and production of sex-inducing substances.

Recently, a new paradigm has been proposed, namely, that besides morphogens and growth factors, various metabolic pathways also constitute important regulatory mechanisms for controlling the somatic and germline stem cell behavior (*e.g*., self-renewal proliferation and differentiation). Metabolic pathways convey information on the changes in the stem cell niche, physiological status, and nutrient availability to reprogram stem cell fate *via* epigenetics; conversely, metabolic pathways are reprogrammed by stem cell factors depending on cellular needs^17^. Nutrient-sensing pathways, such as the insulin signaling pathway and mTOR signaling pathway, play major roles in regulating the metabolic network and stem cell behavior^18,19^. For example, a crucial stem cell regulator mTOR is an evolutionarily conserved kinase that integrates information regarding mitogens, energy, and nutrient (particularly amino acid) levels, and modulates cell division and growth^49–51^. Insulin and insulin-like growth factors also transfer signals to mTOR, and mTOR controls the insulin signaling pathway by regulating several downstream components^52^. In planarians, although the relationship between the insulin and mTOR signaling pathways is yet to be elucidated, several studies on *S. mediterranea* revealed that an insulin-like peptide regulates the proliferation of neoblasts and male germline cells^53^, and that the mTOR signaling pathway is involved in controlling the proliferation of neoblasts during cell turnover and regeneration^54,55^. The KEGG pathway enrichment analysis performed in the current study revealed that, in addition to the differences in amino acid metabolism, the insulin signaling pathway was enriched in the sexual *D. ryukyuensis* (Tables 2 and 4), and that the mTOR signaling pathway was enriched when an expanded set of sexual DEGs was used (*i.e*., DEGs identified using relaxed statistical criteria) (Table 4). Some genes in these pathways (*e.g*., the mTOR homolog gene in *D. ryukyuensis*, TR47902|c0_g1_i1 in the RNA-seq dataset) was not identified as DEG because it was expressed both in asexual and sexual worms. However, the present study suggested that these pathways were probably activated to differentially regulate neoblasts and/or germ cells in sexual worms, and a future detailed examination of gene expression and function using *D. ryukyuensis* should provide insights into how these pathways are fine-tuned in response to “sexuality”.

In the future, the investigation of *D. ryukyuensis* may provide new insights into the mechanism(s) that differentially modulates stem cell behavior *via* a metabolic state that is epigenetically reprogrammed by endogenous sex-inducing substances, rather than daily diet or nutrient availability. Currently, cancer^56,57^ and devastating parasitic diseases caused by flatworms^21^ are considered stem cell diseases. Since serotonin, an ovary-inducing substance identified based on the differentially enriched tryptophan metabolism in the present study, indeed exerts stimulatory (or inhibitory, concentration-dependent) effects on cancer^58^, further knowledge on stem cell control in *D. ryukyuensis* may potentially open up new therapeutic perspectives for such diseases.

## Methods

### Organisms

An exclusively asexual strain of the planarian *D. ryukyuensis*, the OH strain, was established by Dr. S. Ishida at Hirosaki University (Hirosaki, Japan). The OH strain was maintained at 20 °C in autoclaved tap water and fed organic chicken liver (Champool, Kanagawa, Japan). Under these conditions, the OH strain is exclusively fissiparous and has never reproduced sexually, resulting in the establishment of a clonal asexual population. However, the OH strain can be sexualized experimentally, after feeding it the sexual planarian *B. brunnea*^10,14^. In the current study, the OH strain worms were used as the asexual worms, and the experimentally sexualized OH strain worms were used as the sexual worms. Innate sexual offspring were obtained by inbreeding the sexualized worms. Three sexual offspring were selected and a clonal population was established from each, via consecutive amputation and regeneration. For RNA-seq experiments, a mixture of three clones of innate sexual worms was used.

### RNA-Seq

Total RNA was extracted from asexual, sexual, and innate sexual worms, using Sepasol RNA I Super G (Nacalai Tesque, Kyoto, Japan) following the manufacturer’s instructions. Each sample contains 9 worms for the asexual sample; 3 worms for the sexual sample; and 3 worms for the innate sexual samples. Different numbers of worms were used because of differences in body size. Total RNA was treated with TURBO^TM^ DNase using the TURBO DNA-free kit (Thermo Fisher Scientific, Waltham, MA, USA) and was purified using the RNeasy micro kit (QIAGEN, Hilden, Germany), following the manufacturers’ recommendations. RNA integrity was validated using an Agilent 2100 Bioanalyzer (Agilent Technologies, Santa Clara, CA, USA). The cDNA libraries were prepared using the TruSeq RNA sample preparation kit v2 (Illumina, San Diego, CA, USA) following the manufacturer’s instructions (“Low Sample Protocol”). Briefly, mRNA was purified from 0.5 μg of total RNA using oligo-dT magnetic beads, and chemically fragmented. The cDNA was synthesized using SuperScript II reverse transcriptase (Invitrogen, Carlsbad, CA, USA) and random primers. The resultant cDNA was purified using AMPure XP beads (Beckman Coulter, Brea, CA, USA). The cDNA was then subjected to end-repair processing, and the 3′-ends were adenylated and ligated with paired-end adaptors (Illumina). The cDNA fragments were amplified using adaptor-specific primers (Illumina). The enriched cDNA libraries were validated using an Agilent 2100 Bioanalyzer (Agilent Technologies). Multiplex sequencing of paired-end reads was performed using an equimolar mixture of the final cDNA libraries and an Illumina Hiseq. 2000 system (Illumina).

For RNA-seq, five biological replicates were used for each worm type; only four of these replicates were TURBO^TM^ DNase-treated. All five replicates were used for *de novo* assembly to create reference sequences, but only four TURBO^TM^ DNase-treated replicates were used in the subsequent RNA-seq data analysis.

### Raw Data Processing and *De Novo* Assembly

The raw Illumina reads were cleaned up with cutadapt (v1.8.1)^59^. Low-quality ends [quality-value (QV) < 30] and adapter sequences were trimmed. To build a comprehensive set of reference transcript sequences, cleaned reads derived from all libraries (asexual, experimentally-induced sexual, and innate sexual planarians) were pooled and input into the Trinity (v2.0.6)^60^ *de novo* RNA-seq assembler in the paired-end mode using default parameters.

### DEG Identification

Data for the asexual and sexual OH strain were used. RNA-seq data from innate sexual worms were only used to enrich the transcriptome catalogue for future studies, and thus were excluded from the analysis in the present study. The cleaned reads were mapped to a reference transcript set that was created by *de novo* assembly, using Bowtie2 v2.2.6^61^. Mapped reads were counted using eXpress v1.5.1^62^. The read count data was then analyzed to identify DEGs in asexual and sexual worms, using edgeR v3.12.0^63,64^ in R v3.2.2^65^. Transcripts with an FDR < 0.01 were designated as DEGs, unless otherwise stated. Unless otherwise specified, default parameters were used in these analyses.

### Annotation and Enrichment Analysis

All predicted CDSs (57,762 sequences) were searched against the sequences deposited in the National Center for Biotechnology Information non-redundant protein sequence database (ftp://ftp.ncbi.nlm.nih.gov/blast/db/; last accessed 13 February 2016), using BLASTp v2.2.27. The functional information on proteins, including pathway annotation, was obtained by using BlastKOALA^66^ (http://www.kegg.jp/blastkoala). Blast2GO v3.1^67^ was used to detect the associated GO terms, describing biological processes, molecular functions, and cellular components. Enrichment analysis was performed using DAVID Bioinformatics Resources v6.7^68,69^ (https://david-d.ncifcrf.gov/) and the DEG lists. FDR corrections were calculated using the Benjamini-Hochberg procedure^70^.

### Whole-Mount *In Situ* Hybridization

To obtain partial nucleotide sequences used for the design of *in situ* hybridization probes, the target genes were cloned, as previously described^39^. The primers used in the current study are given in Supplementary Table S5. Digoxigenin (DIG)-labeled anti-sense RNA probes were synthesized *in vitro* using DIG-11-UTP (Roche, Mannheim, Germany) and the MEGA script T7 (or SP6) kit (Thermo Fisher Scientific).

Whole-mount *in situ* hybridization was performed as previously described^25^. Three biological replicates were used for each worm type. The protocol was optimized according to planarian sexuality (because of their differences in size), with a 10-min proteinase K treatment of asexual worms and 15-min treatment of sexual worms. DIG-labeled probes were detected with alkaline phosphatase-conjugated anti-DIG antibodies (1:2,000, Roche, cat. no. 11093274910), and the reactions were developed at 20 °C using 170 μg/mL of nitro-blue tetrazolium (Roche) and 175 μg/mL of 5-bromo-4-chloro-3′-indolyphosphate (Roche). Specimens were examined, and images were taken using a digital microscopy setup with an Olympus SZX10 microscope (Olympus Corporation, Tokyo, Japan) and an Olympus DP22 digital camera (Olympus Corporation).

### qRT-PCR

Total RNA was extracted from individual worms and treated with DNase I, as described in the RNA-seq section. About 0.5 μg of total RNA was used to prepare cDNA using the ReverTra Ace kit (Toyobo, Tokyo, Japan). qRT-PCR was performed using the KAPA SYBR Fast qPCR master mix kit (KAPA Biosystems, Wilmington, MA, USA) and a DNA Engine Opticon 2 system (Bio-Rad Laboratories, Hercules, CA, USA) following the manufacturers’ instructions.

In the present study, qRT-PCR was performed to (i) validate some of the top 25 asexual DEGs and top 40 sexual DEGs with the largest log_2_FC, (ii) validate the genes in the amino acid metabolic pathways enriched both in asexual and sexual worms, and (iii) examine the expression levels of marker genes for the reproductive organs in the serotonin feeding assay. Primers used for purposes (i) and (ii) are listed in Supplementary Tables S6 and S4, respectively. For purpose (iii), the following primers were used; *Dr-nanos*, forward, 5′-TTTGGCAATCGGTAACTTCC-3′ and reverse, 5′-CGCAAGCAATGTGAAGTCTG-3′; *DrY1*, forward, 5′-TATGCCTCCACCTCCTCAAG-3′ and reverse, 5′-CGCCACGATAACCCATAATC-3′; and *Dryg*, forward, 5′-AAATCTATCGTTGCCCGATG-3′ and reverse, 5′-TCGCATCGTTTTGATGTTTG-3′. As an internal control, the *D. ryukyuensis* homolog of the gene encoding elongation factor 1 alpha (*Dref1a*, forward, 5′-TTGGTTATCAACCCGATGGTG-3′ and reverse, 5′-TCCCATCCCTTGTACCATGAC-3′) was used^32^ in all cases. The cycling conditions were as follows: 1 min at 95 °C; 40 cycles of 2 s at 95 °C and 30 s at 60 °C; and 1 min at 65 °C. Differences in the obtained threshold cycle (Ct) values between samples were calculated using the *ΔΔ*Ct method. Briefly, ΔCt [where ΔCt = Ct(target gene) - Ct(internal control)] was calculated for each sample (e.g., asexual and sexual), and then ΔΔCt [where ΔΔCt = ΔCt(sample) - the average of ΔCt(calibrator)] was calculated. Calibrators were the sexual worms for purposes (i) and (ii) and the control worms for purpose (iii). Statistical tests were performed on the ΔΔCt values. Relative expression was calculated as 2^−ΔΔCt^.

Statistical tests were performed using R v3.2.2^65^. When gene amplification was not detected, which was often the case for sexual DEGs tested in asexual worms, expression was treated as not available (NA) in the calculations. The Shapiro–Wilk test was used to validate the normal distributions of obtained data, and the F-test or Berlett’s test was used to validate equality of variances; then, Student’s *t*-test was used to compare gene expression levels between asexual and sexual samples [purposes (i) and (ii)]. In a few cases, Welch’s *t*-test was used because of unequal variances between the samples. To compare gene expression levels among the serotonin-fed groups [purpose (iii)], Tukey’s honestly significant difference (HSD) test was used.

### Serotonin Bioassay

Asexual worms were fed freeze-dried chicken liver homogenates that were either supplemented with serotonin hydrochloride (H9523-25MG, Sigma-Aldrich, St. Louis, MO, USA) for the treatment groups or not supplemented for the control group. Three concentrations of serotonin were fed to the worms: 1.2, 12, or 120 ng/worm/d. Feeding took place every day for 4 weeks, as previously described^31^. The worms were kept at a density of 5 worms per 90-mm plastic petri dish, in which fissioning of asexual worms was rarely observed during 4 weeks of everyday feeding.

To evaluate ovarian induction, the treated and control worms were examined under an Olympus SZX10 microscope (Olympus Corporation). In live worms, the ovary is externally visible as a dark-colored point under the microscope (Fig. 4A). At the completion of 3 weeks of feeding, all worms were examined; then, two worms from each treatment group were chosen, and images of live worms were taken using an Olympus DP22 digital camera (Olympus Corporation). All worms were then replaced and treated for one additional week. After 4 weeks of treatment, the presence or absence of induced ovaries in live worms was observed. As the testis and yolk glands are invisible in live worms, 2 worms with induced ovaries were chosen from each treatment group (worms in the control group were randomly chosen since no worms appeared to develop ovaries) for histological analysis to examine the formation of other reproductive organs (see section “Histology”), and 8 different worms were chosen for qRT-PCR analysis (see section “qRT-PCR”). In 8 worms from each treatment group, the body weight of each worm was measured. Briefly, a live worm on a paint brush was quickly dried with a paper towel and was transferred into a tube on a digital scale.

Statistical tests were performed using R v3.2.2^65^. Fisher’s exact test was used for the obtained ovarian induction count data to examine the effect of the serotonin bioassay. Tukey’s HSD test was used to compare the body weight between the control and serotonin-fed groups.

### Histology

After the serotonin bioassay, 2 worms from each treatment group were chosen for histological analysis. Individual worms were relaxed in cold 2% (v/v) HCl in 5/8 Holtfreter’s solution for 5 min and then fixed in 4% paraformaldehyde and 5% methanol in 5/8 Holtfreter’s solution for 3 h at room temperature. The fixed specimens were dehydrated through an ethanol series, cleared in xylene, and embedded in Paraplast Plus embedding medium (Sigma-Aldrich Co., St. Louis, MO, USA). The embedded specimens were cut into 4-µm thick sections and stained with hematoxylin and eosin (HE) using Mayer’s Hematoxylin Solution (Wako, Osaka, Japan) and Eosin Y (yellowish), Certistain® for microscopy (Merck, Darmstadt, Germany). Specimens were examined, and images were taken using a digital microscopy setup involving a Nikon Eclipse E800 microscope (Nikon, Tokyo, Japan) and an Olympus DP22 digital camera (Olympus Corporation).

## Supplementary information

Supplementary Dataset 1

Supplementary Materials

Permission

supplementary dataset 2

supplementary dataset 3

## Data Availability

Illumina sequences generated during the current study are available from the DNA Data Bank of Japan (DDBJ) Sequence Read Archive (DRA, http://trace.ddbj.nig.ac.jp/dra/) under the accession number DRA006043. The *de novo* assembly of the transcripts produced in the present study is available as Supplementary Dataset 1. Predicted CDSs are available as Supplementary Dataset 2. Information of IDs, expression levels (logFC and logCPM), and annotations for all contigs is available as Supplementary Dataset 3.

## References

[1] Hodgson, A. N. In Reproduction and development biology Encyclopedia of Life Support System (ed. A. M. da Silva) 1–27 (Developed under the auspices the UNESCO, EOLSS Publishers, 2009).

[2] Agrawal AF (2006). Evolution of sex: why do organisms shuffle their genotypes?. Curr Biol..

[3] Poulin R, Lagrue C (2015). The ups and downs of life: population expansion and bottlenecks of helminth parasites through their complex life cycle. Parasitology..

[4] Aboobaker AA (2011). Planarian stem cells: a simple paradigm for regeneration. Trends Cell Biol..

[5] Baguñà J (2012). The planarian neoblast: the rambling history of its origin and some current black boxes. Int J Dev Biol..

[6] Egger B, Gschwentner R, Rieger R (2007). Free-living flatworms under the knife: past and present. Dev Genes Evol..

[7] Janssen T (2015). The first multi-gene phylogeny of the Macrostomorpha sheds light on the evolution of sexual and asexual reproduction in basal Platyhelminthes. Mol Phylogenet Evol..

[8] Kobayashi K, Arioka S, Hoshi M (2002). Seasonal changes in the sexualization of the planarian Dugesia ryukyuensis. Zoolog Sci..

[9] Grasso M, Benazzi M (1973). Genetic and physiologic control of fissioning and sexuality in planarians. Journal of Embryology and Experimental Morphology..

[10] Kobayashi K, Koyanagi R, Matsumoto M, Cabrera JP, Hoshi M (1999). Switching from asexual to sexual reproduction in the planarian Dugesia ryukyuensis: Bioassay system and basic description of sexualizing process. Zoological Science..

[11] Sakurai T (1981). Sexual induction by feeding in an asexual strain of the freshwater planarian, Dugesia japonica japonica. Annot Zool Jap..

[12] Nakagawa, H. *et al*. A comprehensive comparison of sex-inducing activity in asexual worms of the planarian Dugesia ryukyuensis: the crucial sex-inducing substance appears to be present in yolk glands in Tricladida. *Zoological Letters*. **4**, 10.1186/s40851-018-0096-9 (2018). 10.1186/s40851-018-0096-9 PMC5996458 29942643

[13] Wagner DE, Wang IE, Reddien PW (2011). Clonogenic neoblasts are pluripotent adult stem cells that underlie planarian regeneration. Science..

[14] Kobayashi K, Hoshi M (2002). Switching from asexual to sexual reproduction in the planarian Dugesia ryukyuensis: change of the fissiparous capacity along with the sexualizing process. Zoolog Sci..

[15] Baguñà J (1999). From morphology and karyology to molecules. New methods for taxonomical identification of asexual populations of freshwater planarians. A tribute to Professor Mario Benazzi. Italian Journal of Zoology..

[16] Newmark PA, Sanchez Alvarado A (2002). Not your father’s planarian: a classic model enters the era of functional genomics. Nat Rev Genet..

[17] Shyh-Chang N, Ng HH (2017). The metabolic programming of stem cells. Genes Dev..

[18] Ables ET, Laws KM, Drummond-Barbosa D (2012). Control of adult stem cells *in vivo* by a dynamic physiological environment: diet-dependent systemic factors in Drosophila and beyond. Wiley Interdiscip Rev Dev Biol..

[19] Fukuyama, M. In Reproductive and Developmental Strategies: The Continuity of Life (eds Kazuya Kobayashi, Takeshi Kitano, Yasuhiro Iwao, & Mariko Kondo) 69–101 (Springer Japan, 2018).

[20] Egger B (2015). A transcriptomic-phylogenomic analysis of the evolutionary relationships of flatworms. Curr Biol..

[21] Wendt GR, Collins JJ (2016). Schistosomiasis as a disease of stem cells. Current Opinion in Genetics &. Development..

[22] Collins JJ (2013). Adult somatic stem cells in the human parasite Schistosoma mansoni. Nature..

[23] Ramm SA (2017). Exploring the sexual diversity of flatworms: Ecology, evolution, and the molecular biology of reproduction. Molecular Reproduction and Development..

[24] Wang B, Collins JJ, Newmark PA (2013). Functional genomic characterization of neoblast-like stem cells in larval Schistosoma mansoni. eLife..

[25] Kobayashi K, Maezawa T, Nakagawa H, Hoshi M (2012). Existence of two sexual races in the planarian species switching between asexual and sexual reproduction. Zoolog Sci..

[26] Kanehisa M, Furumichi M, Tanabe M, Sato Y, Morishima K (2017). KEGG: new perspectives on genomes, pathways, diseases and drugs. Nucleic Acids Res.

[27] Kanehisa M, Goto S (2000). KEGG: kyoto encyclopedia of genes and genomes. Nucleic Acids Res.

[28] Kanehisa M, Sato Y, Kawashima M, Furumichi M, Tanabe M (2016). KEGG as a reference resource for gene and protein annotation. Nucleic Acids Res.

[29] Kobayashi K (2017). The identification of D-tryptophan as a bioactive substance for postembryonic ovarian development in the planarian Dugesia ryukyuensis. Sci Rep..

[30] Nakagawa H, Ishizu H, Chinone A, Kobayashi K, Matsumoto M (2012). The Dr-nanos gene is essential for germ cell specification in the planarian Dugesia ryukyuensis. Int J Dev Biol.

[31] Kobayashi K, Hoshi M (2011). Sex-inducing effect of a hydrophilic fraction on reproductive switching in the planarian Dugesia ryukyuensis (Seriata, Tricladida). Frontiers in Zoology..

[32] Ishizuka H (2007). The Dugesia ryukyuensis database as a molecular resource for studying switching of the reproductive system. Zoolog Sci..

[33] Salvetti A (2002). Characterization of DeY1, a novel Y-box gene specifically expressed in differentiating male germ cells of planarians. Gene Expression Patterns.

[34] Wang Y, Stary JM, Wilhelm JE, Newmark PA (2010). A functional genomic screen in planarians identifies novel regulators of germ cell development. Genes Dev.

[35] Hase S, Kobayashi K, Koyanagi R, Hoshi M, Matsumoto M (2003). Transcriptional pattern of a novel gene, expressed specifically after the point-of-no-return during sexualization, in planaria. Dev Genes Evol..

[36] Martín-Durán JM, Monjo F, Romero R (2012). Planarian embryology in the era of comparative developmental biology. Int J Dev Biol..

[37] Sluys, R. A Monograph of the Marine Triclads. (CRC Press, 2017).

[38] Counts JT, Hester TM, Rouhana L (2017). Genetic expansion of chaperonin-containing TCP-1 (CCT/TRiC) complex subunits yields testis-specific isoforms required for spermatogenesis in planarian flatworms. Mol Reprod Dev.

[39] Maezawa T (2014). Planarian D-amino acid oxidase is involved in ovarian development during sexual induction. Mech Dev..

[40] Maezawa, T., Sekii, K., Ishikawa, M., Okamoto, H. & Kobayashi, K. In Reproductive and Developmental Strategies: The Continuity of Life (eds Kazuya Kobayashi, Takeshi Kitano, Yasuhiro Iwao, & Mariko Kondo) 175–201 (Springer Japan, 2018).

[41] Tinikul Y, Joffre Mercier A, Soonklang N, Sobhon P (2008). Changes in the levels of serotonin and dopamine in the central nervous system and ovary, and their possible roles in the ovarian development in the giant freshwater prawn, Macrobrachium rosenbergii. Gen Comp Endocrinol..

[42] Stepinska U, Kuwana T, Olszanska B (2015). Serotonin receptors are selectively expressed in the avian germ cells and early embryos. Zygote..

[43] Buznikov GA, Nikitina LA, Galanov A, Malchenko LA, Trubnikova OB (1993). The control of oocyte maturation in the starfish and amphibians by serotonin and its antagonists. Int J Dev Biol..

[44] Bodis J (1993). Relationship between the monoamine and gonadotropin content in follicular fluid of preovulatory graafian follicles after superovulation treatment. Exp Clin Endocrinol..

[45] Sato K (2006). Identification and origin of the germline stem cells as revealed by the expression of nanos-related gene in planarians. Dev Growth Differ.

[46] Nishimura K (2007). Identification and distribution of tryptophan hydroxylase (TPH)-positive neurons in the planarian Dugesia japonica. Neurosci Res..

[47] Chong T, Stary JM, Wang Y, Newmark PA (2011). Molecular markers to characterize the hermaphroditic reproductive system of the planarian Schmidtea mediterranea. BMC Dev Biol.

[48] Rouhana L, Tasaki J, Saberi A, Newmark PA (2017). Genetic dissection of the planarian reproductive system through characterization of Schmidtea mediterranea CPEB homologs. Dev Biol.

[49] Zoncu R, Efeyan A, Sabatini DM (2011). mTOR: from growth signal integration to cancer, diabetes and ageing. Nat Rev Mol Cell Biol..

[50] Russell RC, Fang C, Guan KL (2011). An emerging role for TOR signaling in mammalian tissue and stem cell physiology. Development..

[51] Goberdhan DC, Wilson C, Harris AL (2016). Amino acid sensing by mTORC1: intracellular transporters mark the spot. Cell Metab..

[52] Yoon Mee-Sup (2017). The Role of Mammalian Target of Rapamycin (mTOR) in Insulin Signaling. Nutrients.

[53] Miller CM, Newmark PA (2012). An insulin-like peptide regulates size and adult stem cells in planarians. Int J Dev Biol..

[54] Peiris TH (2012). TOR signaling regulates planarian stem cells and controls localized and organismal growth. J Cell Sci..

[55] Tu KC, Pearson BJ, Alvarado AS (2012). TORC1 is required to balance cell proliferation and cell death in planarians. Developmental biology..

[56] Blacking TM, Wilson H, Argyle DJ (2007). Is cancer a stem cell disease? Theory, evidence and implications. Vet Comp Oncol..

[57] Tu SM (2013). Cancer: a “stem-cell” disease?. Cancer Cell Int..

[58] Sarrouilhe D, Clarhaut J, Defamie N, Mesnil M (2015). Serotonin and cancer: what is the link?. Curr Mol Med..

[59] Martin M (2011). Cutadapt removes adapter sequences from high-throughput sequencing reads. EMBnet.journal..

[60] Grabherr MG (2011). Full-length transcriptome assembly from RNA-Seq data without a reference genome. Nat Biotechnol..

[61] Langmead B, Salzberg SL (2012). Fast gapped-read alignment with Bowtie 2. Nat Methods..

[62] Roberts A, Pachter L (2013). Streaming fragment assignment for real-time analysis of sequencing experiments. Nat Methods..

[63] McCarthy DJ, Chen Y, Smyth GK (2012). Differential expression analysis of multifactor RNA-Seq experiments with respect to biological variation. Nucleic Acids Res..

[64] Robinson MD, McCarthy DJ, Smyth G (2010). K. edgeR: a Bioconductor package for differential expression analysis of digital gene expression data. Bioinformatics..

[65] R: A language and environment for statistical computing (R Development Core Team, R Foundation for Statistical Computing, Vienna, Austria, 2004).

[66] Kanehisa M, Sato Y, Morishima K (2016). BlastKOALA and GhostKOALA: KEGG tools for functional characterization of genome and metagenome sequences. J Mol Biol..

[67] Conesa A (2005). Blast2GO: a universal tool for annotation, visualization and analysis in functional genomics research. Bioinformatics..

[68] Huang DW, Sherman BT, Lempicki RA (2009). Bioinformatics enrichment tools: paths toward the comprehensive functional analysis of large gene lists. Nucleic Acids Res..

[69] Huang DW, Sherman BT, Lempicki RA (2009). Systematic and integrative analysis of large gene lists using DAVID bioinformatics resources. Nature Protocols..

[70] Benjamini Y, Hochberg Y (1995). Controlling the false discovery rate: a practical and powerful approach to multiple testing. Journal of the Royal Statistical Society. Series B (Methodological)..

