# Author Correction: Transcriptomic analysis reveals differences in the regulation of amino acid metabolism in asexual and sexual planarians

**DOI:** 10.1038/s41598-019-48084-6

**Published:** 2019-08-08

**Authors:** Kiyono Sekii, Shunta Yorimoto, Hikaru Okamoto, Nanna Nagao, Takanobu Maezawa, Yasuhisa Matsui, Katsushi Yamaguchi, Ryohei Furukawa, Shuji Shigenobu, Kazuya Kobayashi

**Affiliations:** 1 Department of Biology, Faculty of Agriculture and Life Science, Hirosaki University, 3 Bunkyo-cho, Hirosaki, Aomori 036-8561 Japan; 2 Department of Integrated Science and Technology, National Institute of Technology, Tsuyama College, 624-1 Numa, Tsuyama, Okayama 708–8509 Japan; 3 Cell Resource Center for Biomedical Research, Institute of Development, Aging and Cancer, Tohoku University, 4-1 Seiryo-machi, Sendai, 980-8575 Japan; 4 NIBB Core Research Facilities, National Institute for Basic Biology, 38 Nishigonaka Myodaiji, Okazaki, 444-8585 Japan; 5 Division of Biomedical Information Analysis, Iwate Tohoku Medical Megabank Organization, Iwate Medical University, 2-1-1 Nishitokuda, Yanaba-cho, Shiwa-gun, Iwate 028-3694 Japan; 6 Department of Biology, Research and Education Center for Natural Sciences, Keio University, 4-1-1 Hiyoshi, Kohoku-ku, Yokohama, Kanagawa 223-8521 Japan; 7 Department of Basic Biology, School of Life Science, SOKENDAI (The Graduate University for Advanced Studies), 38 Nishigonaka Myodaiji, Okazaki, 444-8585 Japan

Correction to: *Scientific Reports* 10.1038/s41598-019-42025-z, published online 16 April 2019

Supplementary Datasets S2 and S3 were omitted from the original version of this Article. This has been corrected in the HTML version of the Article; the PDF version was correct at time of publication.

