# Supplementary material for: Transcriptomic analysis reveals differences in the regulation of amino acid metabolism in asexual and sexual planarians
Source: Sci Rep. 2019 Apr 16;9:6132. doi: 10.1038/s41598-019-42025-z (PMC6467871; doi:10.1038/s41598-019-42025-z)
Supplement: Supplementary file 2 — Supplementary Materials [file 41598_2019_42025_MOESM2_ESM.pdf]

Title: **Transcriptomic analysis reveals differences in the regulation of amino acid metabolism in asexual and sexual planarians**

Authors: Kiyono Sekii[a,†], Shunta Yorimoto[a,†], Hikaru Okamoto[a], Nanna Nagao[a], Takanobu Maezawa[b], Yasuhisa Matsui[c], Katsushi Yamaguchi[d], Ryohei Furukawa[e,f,*], Shuji Shigenobu[d,g*] & Kazuya Kobayashi[a,*]

[†] These authors contributed equally to this work.

Author affiliations:

[a] Department of Biology, Faculty of Agriculture and Life Science, Hirosaki University, 3 Bunkyo-cho, Hirosaki, Aomori 036-8561, Japan.

[b] Department of Integrated Science and Technology, National Institute of Technology, Tsuyama College, 624-1 Numa, Tsuyama, Okayama 708–8509, Japan.

[c] Cell Resource Center for Biomedical Research, Institute of Development, Aging and Cancer, Tohoku University, 4-1 Seiryo-machi, Sendai 980-8575, Japan.

[d] NIBB Core Facility, National Institute for Basic Biology, 38 Nishigonaka Myodaiji, Okazaki 444-8585, Japan

[e] Division of Biomedical Information Analysis, Iwate Tohoku Medical Megabank Organization, Iwate Medical University, 2-1-1 Nishitokuda, Yanaba-cho, Shiwa-gun, Iwate 028-3694, Japan.

[f] Department of Biology, Research and Education Center for Natural Sciences, Keio University, 4-1-1 Hiyoshi, Kohoku-ku, Yokohama, Kanagawa 223-8521, Japan.

[g] Department of Basic Biology, School of Life Science, SOKENDAI (The Graduate University for Advanced Studies), 38 Nishigonaka Myodaiji, Okazaki 444-8585, Japan.

[*] ***Co-corresponding authors***:

Kazuya Kobayashi

Hirosaki University, Department of Biology, Faculty of Agriculture and Life Science, Bunkyo-cho 3, 036-8224 Aomori, Japan

Tel: ++81-172-39-3587

Email: kobkyram@hirosaki-u.ac.jp

Shuji Shigenobu
NIBB Core Facility, National Institute for Basic Biology, 38 Nishigonaka Myodaiji, Okazaki 444-8585, Japan
Tel: +81-564-55-7670
Email: shige@nibb.ac.jp

Ryohei Furukawa
Keio University, Department of Biology, Research and Education Center for Natural Sciences, 4-1-1 Hiyoshi, Kohoku-ku, Yokohama, Kanagawa 223-8521, Japan
Tel: +81-45-566-1340
Email: furukawa@keio.jp

***Supplementary materials*:**

Fig. S1: Differences in gene expression between the asexual and sexual modes of reproduction

Legend of Fig. S1

Fig. S2: Qualitative and quantitative validation of asexual DEGs

Legend of Fig. S2

Fig. S3: KEGG pathway mapping of the glycine, serine, and threonine metabolism

Legend of Fig. S3

Fig. S4: KEGG pathway mapping of the arginine and proline metabolism

Legend of Fig. S4

Fig. S5: Additional information on the worms in the serotonin feeding experiment

Legend of Fig. S5

Table. S1: Summary of RNA-seq, *de novo* assembly, and mapping statistics

Table. S2: GO enrichment analysis of asexual and sexual DEGs

Table. S3: DEGs in the enriched KEGG pathways

Table. S4: Detail information of the DEGs in three amino acid metabolic pathways enriched both in asexual and sexual worms; Tryptophan metabolism; Glycine, serine, and threonine metabolism; and Arginine and proline metabolism

Table. S5: Primer sets used for the synthesis of whole-mount *in situ* hybridization probes

Table. S6: Primer sets used for qRT-PCR

Fig. S1

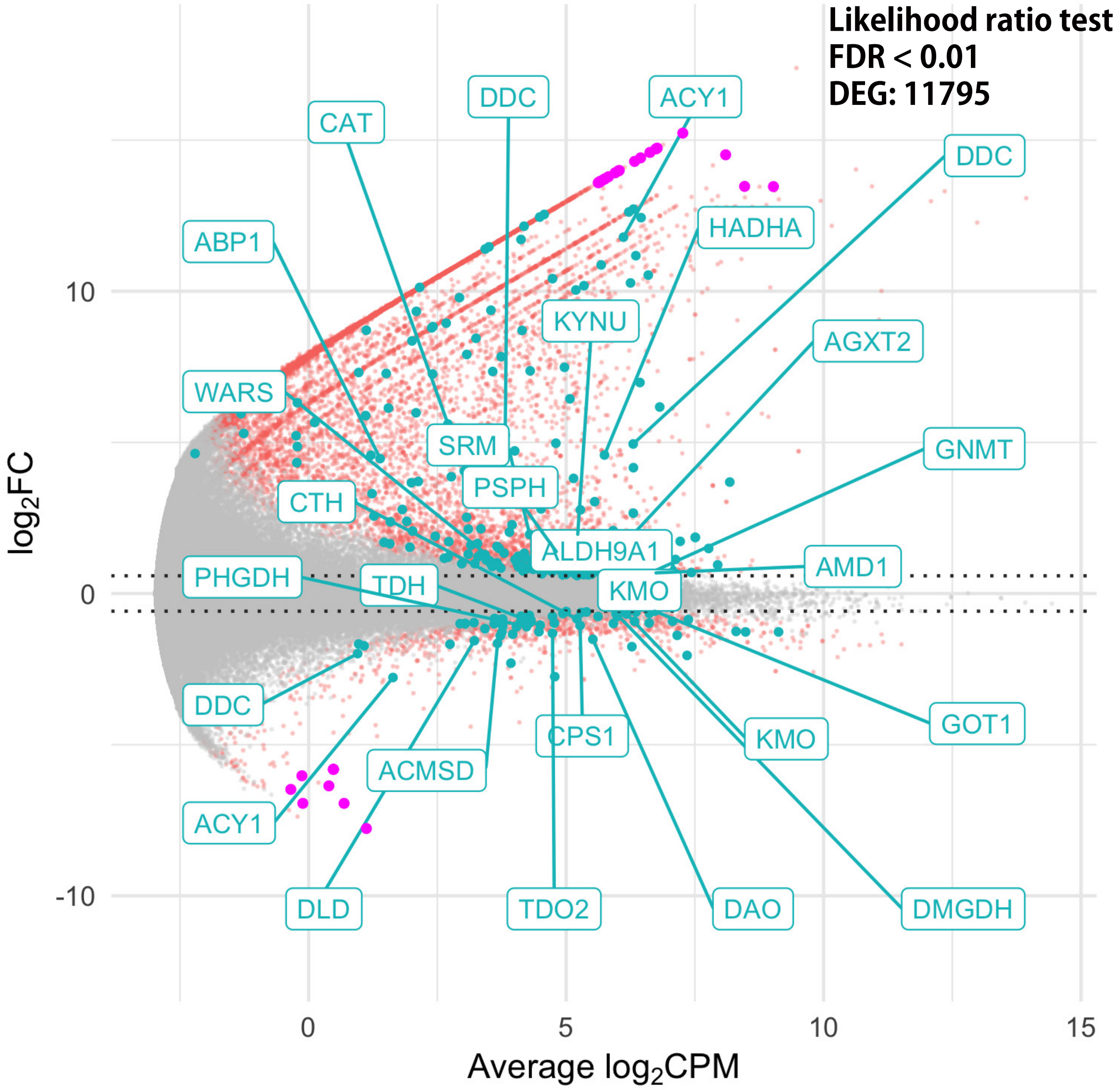

Likelihood ratio test
FDR < 0.01
DEG: 11795
DDC
CAT
ACY1
DDC
ABP1
HADHA
KYNU
AGXT2
WARS
SRM
GNMT
CTH
PSPH
ALDH9A1
AMD1
PHGDH
TDH
KMO
DDC
CPS1
KMO
GOT1
ACMSD
ACY1
DLD
TDO2
DAO
DMGDH
10
0
-10
0
5
10
15
log2FC
Average log2CPM

Supplementary Figure S1. Differences in gene expression between the asexual and sexual modes of reproduction. The sexual to asexual expression ratios (log_2FC sexual/asexual) are plotted against the average expression intensity (log_2CPM; CPM counts per million), using the edgeR v3.12.0[63,64] in R v3.2.2[70]. Asexual ($log_2FC < 0$) and sexual ($log_2FC > 0$) DEGs are indicated in red. The 7 asexual DEGs and 21 sexual DEGs examined for whole-mount *in situ* hybridization and qRT-PCR are indicated in pink. The DEGs from the enriched KEGG pathways are indicated in green. Especially, the DEGs with a KEGG gene name in green are from three of the amino acid metabolic pathways enriched both in asexual and sexual worms: tryptophan metabolism; glycine, serine, and threonine metabolism; and arginine and proline metabolism. The plot was generated based on four biological replicates for each worm type (see RNA-seq section in the Materials and Methods).

Fig. S2

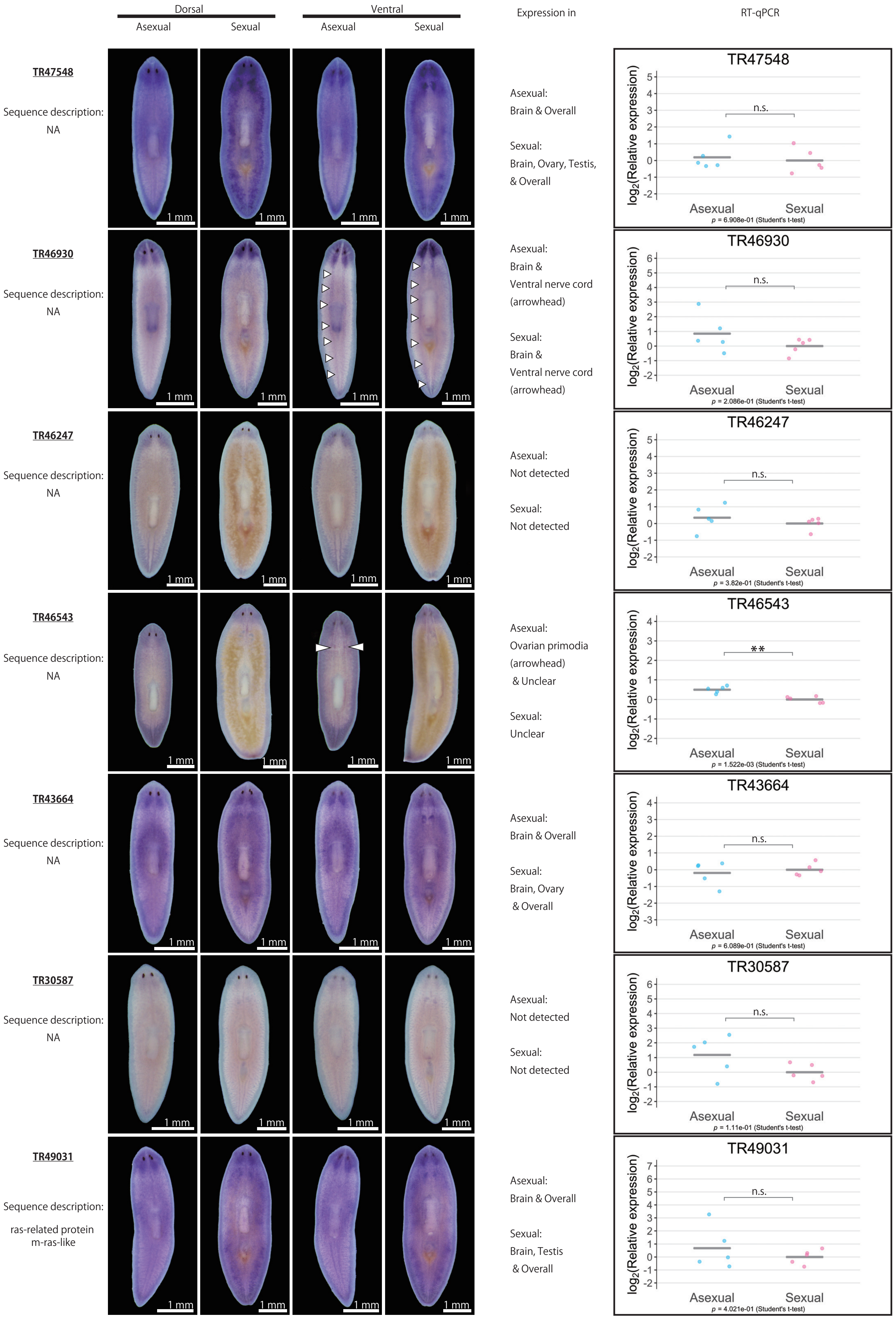
Dorsal
Ventral
Asexual
Sexual
Expression in
RT-qPCR
TR47548
Sequence description:
NA
Asexual:
Brain & Overall
Sexual:
Brain, Ovary, Testis, & Overall
TR46930
Sequence description:
NA
Asexual:
Brain & Ventral nerve cord (arrowhead)
Sexual:
Brain & Ventral nerve cord (arrowhead)
TR46247
Sequence description:
NA
Asexual:
Not detected
Sexual:
Not detected
TR46543
Sequence description:
NA
Asexual:
Ovarian primodia (arrowhead) & Unclear
Sexual:
Unclear
TR43664
Sequence description:
NA
Asexual:
Brain & Overall
Sexual:
Brain, Ovary & Overall
TR30587
Sequence description:
NA
Asexual:
Not detected
Sexual:
Not detected
TR49031
Sequence description:
ras-related protein m-ras-like
Asexual:
Brain & Overall
Sexual:
Brain, Testis & Overall
1 mm
log2(Relative expression)
n.s.
**
p = 6.908e-01 (Student's t-test)
p = 2.086e-01 (Student's t-test)
p = 3.82e-01 (Student's t-test)
p = 1.522e-03 (Student's t-test)
p = 6.089e-01 (Student's t-test)
p = 1.11e-01 (Student's t-test)
p = 4.021e-01 (Student's t-test)

Supplementary Figure S2. Qualitative and quantitative validation of asexual DEGs. Representative whole-mount *in situ* hybridization patterns for the ventral and dorsal sides of worm are shown. The expression pattern was judged based on three replicates. Signals were seen as blue/purple staining. The white arrowheads indicate ovarian primordia. The qRT-PCR data are shown relative to the expression level in the sexual worm, and $\log_2$ (relative expression) on the vertical axis indicates –ΔΔCt. Each circle indicates an individual asexual or sexual worm. Five replicates were used, but data are not shown if the expression was too low to be detected (handled as NA). The bars in the plots indicate the averages of –ΔΔCt. Asterisks indicate significant differences between the asexual and sexual worms (Student's or Welch's *t*-test: * $P < 0.05$; ** $P < 0.01$; *** $P < 0.001$; n.s., not significant).

Fig. S3

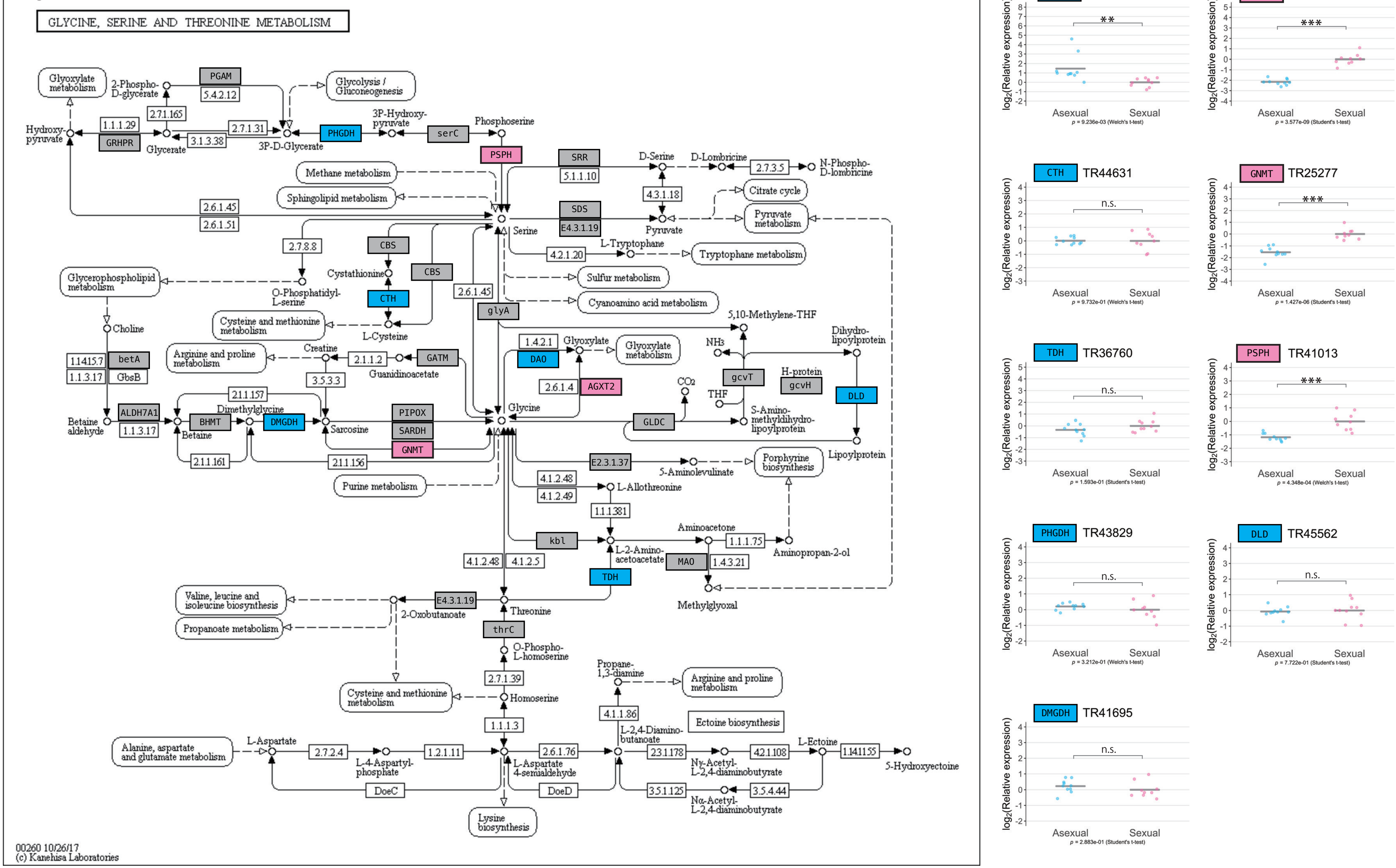

GLYCINE, SERINE AND THREONINE METABOLISM
Glyoxylate metabolism
2-Phospho-D-glycerate
PGAM
5.4.2.12
Glycolysis / Gluconeogenesis
2.7.1.165
1.1.1.29
GRHPR
Hydroxy-pyruvate
Glycerate
3.1.3.38
2.7.1.31
3P-D-Glycerate
PHGDH
3P-Hydroxy-pyruvate
serC
Phosphoserine
PSPH
SRR
5.1.1.10
D-Serine
D-Lombricine
2.7.3.5
N-Phospho-D-lombricine
Methane metabolism
Sphingolipid metabolism
4.3.1.18
Citrate cycle
2.6.1.45
2.6.1.51
SDS
E4.3.1.19
Pyruvate
Pyruvate metabolism
Serine
2.7.8.8
CBS
L-Tryptophane
4.2.1.20
Tryptophane metabolism
Glycerophospholipid metabolism
O-Phosphatidyl-L-serine
Cystathionine
CBS
Sulfur metabolism
CTH
2.6.1.45
Cyanoamino acid metabolism
glyA
Cysteine and methionine metabolism
L-Cysteine
5,10-Methylene-THF
Choline
Arginine and proline metabolism
Creatine
2.1.1.2
GATM
1.4.2.1
Glyoxylate
Glyoxylate metabolism
NH3
Dihydro-lipoylprotein
1.14.15.7
betA
1.1.3.17
GbsB
3.5.3.3
Guanidinoacetate
DAO
gcvT
H-protein
gcvH
2.6.1.4
AGXT2
CO2
THF
DLD
2.1.1.157
Dimethylglycine
Glycine
S-Amino-methyldihydro-lipoylprotein
ALDH7A1
PIPOX
Betaine aldehyde
BHMT
DMGDH
GLDC
1.1.3.17
Betaine
Sarcosine
SARDH
GNMT
Lipoylprotein
2.1.1.161
2.1.1.156
E2.3.1.37
5-Aminolevulinate
Porphyrine biosynthesis
Purine metabolism
4.1.2.48
4.1.2.49
L-Allothreonine
1.1.1.381
Aminoacetone
kbl
1.1.1.75
Aminopropan-2-ol
L-2-Amino-acetoacetate
4.1.2.48
4.1.2.5
MAO
1.4.3.21
TDH
Methylglyoxal
Valine, leucine and isoleucine biosynthesis
E4.3.1.19
Threonine
2-Oxobutanoate
Propanoate metabolism
thrC
O-Phospho-L-homoserine
2.7.1.39
Propane-1,3-diamine
Arginine and proline metabolism
Cysteine and methionine metabolism
Homoserine
4.1.1.86
Ectoine biosynthesis
L-2,4-Diamino-butanoate
1.1.1.3
Alanine, aspartate and glutamate metabolism
L-Aspartate
2.7.2.4
1.2.1.11
2.6.1.76
2.3.1.178
4.2.1.108
L-Ectoine
1.14.11.55
5-Hydroxyectoine
L-4-Aspartyl-phosphate
L-Aspartate 4-semialdehyde
Nγ-Acetyl-L-2,4-diaminobutyrate
DoeC
DoeD
3.5.1.125
3.5.4.44
Nα-Acetyl-L-2,4-diaminobutyrate
Lysine biosynthesis
00260 10/26/17
(c) Kanehisa Laboratories
DAO TR8800
log2(Relative expression)
**
Asexual Sexual
p = 9.236e-03 (Welch's t-test)
AGXT2 TR43837
***
Asexual Sexual
p = 3.577e-09 (Student's t-test)
CTH TR44631
n.s.
Asexual Sexual
p = 9.732e-01 (Welch's t-test)
GNMT TR25277
***
Asexual Sexual
p = 1.427e-06 (Student's t-test)
TDH TR36760
n.s.
Asexual Sexual
p = 1.593e-01 (Student's t-test)
PSPH TR41013
***
Asexual Sexual
p = 4.348e-04 (Welch's t-test)
PHGDH TR43829
n.s.
Asexual Sexual
p = 3.212e-01 (Welch's t-test)
DLD TR45562
n.s.
Asexual Sexual
p = 7.722e-01 (Student's t-test)
DMGDH TR41695
n.s.
Asexual Sexual
p = 2.883e-01 (Student's t-test)

Supplementary Figure S3. KEGG pathway mapping of the glycine, serine, and threonine metabolism. Annotated genes in the RNA-seq analysis were mapped against KEGG pathway maps (www.kegg.jp/kegg/kegg1.html)[26-28] using a KEGG mapper tool (http://www.kegg.jp/kegg/tool/map_pathway2.html). Cyan indicates genes identified as asexual DEGs, and pink indicates genes identified as sexual DEGs. Gray indicates genes not identified as DEGs but expressed in the planarian transcriptome in the present study. The qRT-PCR data for each DEG are shown relative to the expression level in the sexual worm, and $\log_2$ (relative expression) on the vertical axis indicates –ΔΔCt. Each circle indicates an individual asexual or sexual worm. Eight to ten replicates were used, but data are not shown if the expression was too low to be detected or in the case of outliers (handled as NA). The bars in the plots indicate the averages of –ΔΔCt. Asterisks indicate significant differences between the asexual and sexual worms (Student's or Welch's *t*-test: * $P < 0.05$; ** $P < 0.01$; *** $P < 0.001$; n.s., not significant).

Fig. S4

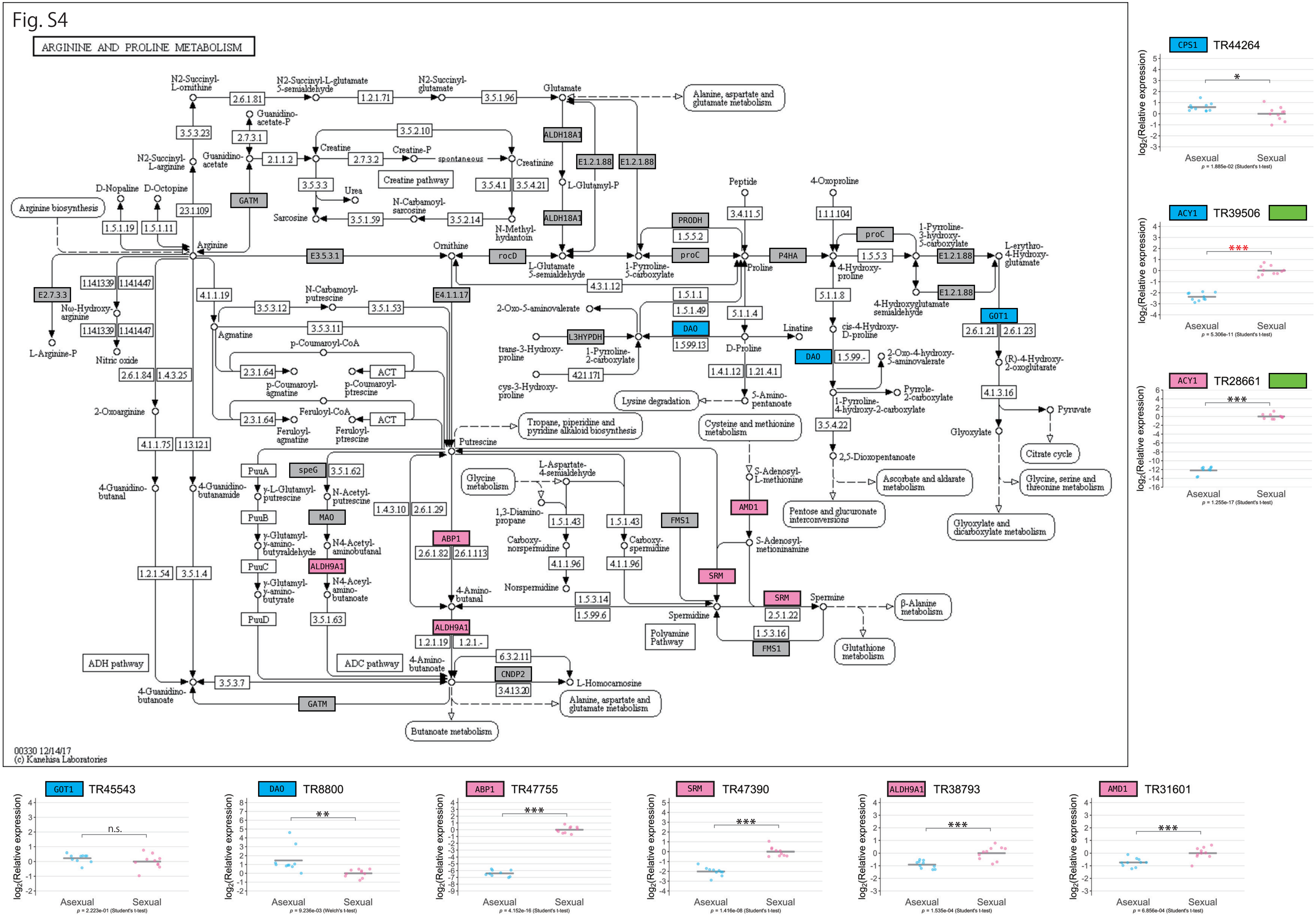
ARGININE AND PROLINE METABOLISM
N2-Succinyl-L-ornithine
N2-Succinyl-L-glutamate 5-semialdehyde
N2-Succinyl-glutamate
Glutamate
Alanine, aspartate and glutamate metabolism
Guanidino-acetate-P
N2-Succinyl-L-arginine
Guanidino-acetate
Creatine
Creatine-P
spontaneous
Creatinine
Creatine pathway
Urea
N-Carbamoyl-sarcosine
Sarcosine
N-Methyl-hydantoin
L-Glutamyl-P
Peptide
4-Oxoproline
D-Nopaline
D-Octopine
Arginine biosynthesis
Arginine
Ornithine
L-Glutamate 5-semialdehyde
1-Pyrroline-5-carboxylate
Proline
4-Hydroxy-proline
1-Pyrroline-3-hydroxy-5-carboxylate
L-erythro-4-Hydroxy-glutamate
4-Hydroxyglutamate semialdehyde
Nω-Hydroxy-arginine
L-Arginine-P
Nitric oxide
N-Carbamoyl-putrescine
Agmatine
p-Coumaroyl-CoA
2-Oxo-5-aminovalerate
trans-3-Hydroxy-proline
1-Pyrroline-2-carboxylate
cys-3-Hydroxy-proline
D-Proline
Linatine
cis-4-Hydroxy-D-proline
2-Oxo-4-hydroxy-5-aminovalerate
(R)-4-Hydroxy-2-oxoglutarate
p-Coumaroyl-agmatine
p-Coumaroyl-ptrescine
Feruloyl-CoA
Feruloyl-agmatine
Feruloyl-ptrescine
2-Oxoarginine
Lysine degradation
5-Amino-pentanoate
Pyrrole-2-carboxylate
1-Pyrroline-4-hydroxy-2-carboxylate
Pyruvate
Tropane, piperidine and pyridine alkaloid biosynthesis
Cysteine and methionine metabolism
Glyoxylate
Putrescine
2,5-Dioxopentanoate
Citrate cycle
Glycine metabolism
L-Aspartate-4-semialdehyde
S-Adenosyl-L-methionine
Ascorbate and aldarate metabolism
Glycine, serine and threonine metabolism
4-Guanidino-butanal
4-Guanidino-butanamide
γ-L-Glutamyl-putrescine
N-Acetyl-putrescine
1,3-Diamino-propane
Pentose and glucuronate interconversions
Glyoxylate and dicarboxylate metabolism
γ-Glutamyl-γ-amino-butyraldehyde
N4-Acetyl-aminobutanal
Carboxy-norspermidine
Carboxy-spermidine
S-Adenosyl-metioninamine
Norspermidine
γ-Glutamyl-γ-amino-butyrate
N4-Acyl-amino-butanoate
4-Amino-butanal
Spermidine
Spermine
β-Alanine metabolism
Polyamine Pathway
Glutathione metabolism
ADH pathway
ADC pathway
4-Amino-butanoate
L-Homocarnosine
4-Guanidino-butanoate
Alanine, aspartate and glutamate metabolism
Butanoate metabolism
2.6.1.81
1.2.1.71
3.5.1.96
3.5.3.23
2.7.3.1
2.1.1.2
3.5.2.10
2.7.3.2
3.5.3.3
3.5.4.1
3.5.4.21
3.5.1.59
3.5.2.14
2.3.1.109
1.5.1.19
1.5.1.11
ALDH18A1
E1.2.1.88
GATM
PRODH
1.5.5.2
3.4.11.5
1.1.1.104
proC
1.5.5.3
E3.5.3.1
rocD
P4HA
E1.2.1.88
1.14.13.39
1.14.14.47
E2.7.3.3
4.1.1.19
3.5.3.12
3.5.1.53
E4.1.1.17
4.3.1.12
1.5.1.1
1.5.1.49
5.1.1.4
5.1.1.8
GOT1
2.6.1.21
2.6.1.23
3.5.3.11
L3HYPDH
DAO
1.5.99.13
1.5.99.-
2.6.1.84
1.4.3.25
2.3.1.64
ACT
4.2.1.171
1.4.1.12
1.21.4.1
4.1.3.16
3.5.4.22
4.1.1.75
1.13.12.1
PuuA
speG
3.5.1.62
AMD1
1.4.3.10
2.6.1.29
PuuB
MAO
1.5.1.43
FMS1
ABP1
2.6.1.82
2.6.1.113
4.1.1.96
SRM
1.2.1.54
3.5.1.4
PuuC
ALDH9A1
1.5.3.14
1.5.99.6
2.5.1.22
PuuD
3.5.1.63
1.2.1.19
1.2.1.-
1.5.3.16
6.3.2.11
CNDP2
3.4.13.20
3.5.3.7
00330 12/14/17
(c) Kanehisa Laboratories
CPS1 TR44264
log2(Relative expression)
Asexual
Sexual
*
p = 1.885e-02 (Student's t-test)
ACY1 TR39506
***
p = 5.306e-11 (Student's t-test)
ACY1 TR28661
***
p = 1.255e-17 (Student's t-test)
GOT1 TR45543
n.s.
p = 2.223e-01 (Student's t-test)
DAO TR8800
**
p = 9.236e-03 (Welch's t-test)
ABP1 TR47755
***
p = 4.152e-16 (Student's t-test)
SRM TR47390
***
p = 1.416e-08 (Student's t-test)
ALDH9A1 TR38793
***
p = 1.535e-04 (Student's t-test)
AMD1 TR31601
***
p = 6.856e-04 (Student's t-test)

Supplementary Figure S4. KEGG pathway mapping of arginine and proline metabolism. Annotated genes in the RNA-seq analysis were mapped against KEGG pathway maps (www.kegg.jp/kegg/kegg1.html)[26-28] using a KEGG mapper tool (http://www.kegg.jp/kegg/tool/map_pathway2.html). Cyan indicates genes identified as asexual DEGs, and pink indicates genes identified as sexual DEGs. Gray indicates genes not identified as DEGs but expressed in the planarian transcriptome in the present study. The qRT-PCR data for each DEG are shown relative to the expression level in the sexual worm, and $\log_2$ (relative expression) on the vertical axis indicates $-\Delta\Delta Ct$. Each circle indicates an individual asexual or sexual worm. Eight to ten replicates were used, but data are not shown if the expression was too low to be detected or in the case of outliers (handled as NA). The bars in the plots indicate the averages of $-\Delta\Delta Ct$. Asterisks indicate significant differences between the asexual and sexual worms (Student's or Welch's *t*-test: * $P < 0.05$; ** $P < 0.01$; *** $P < 0.001$; n.s., not significant); asterisks in red for TR39506 indicate that it was identified as an asexual DEG, but its expression was significantly higher in the sexual worms. Note that TR44264, TR39506, and TR28661 are not shown in the present map because these were mapped to the KEGG pathway map of "arginine biosynthesis", but the results of qRT-PCR are shown on the right side.

Fig. S5

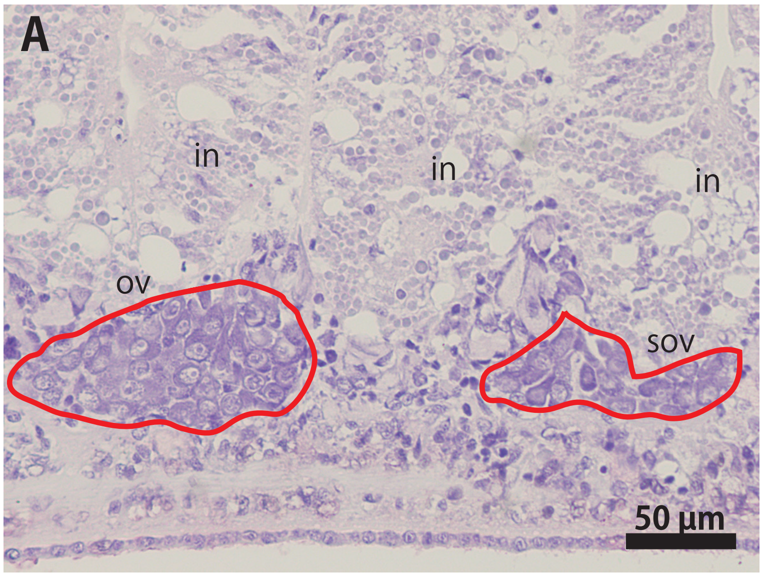

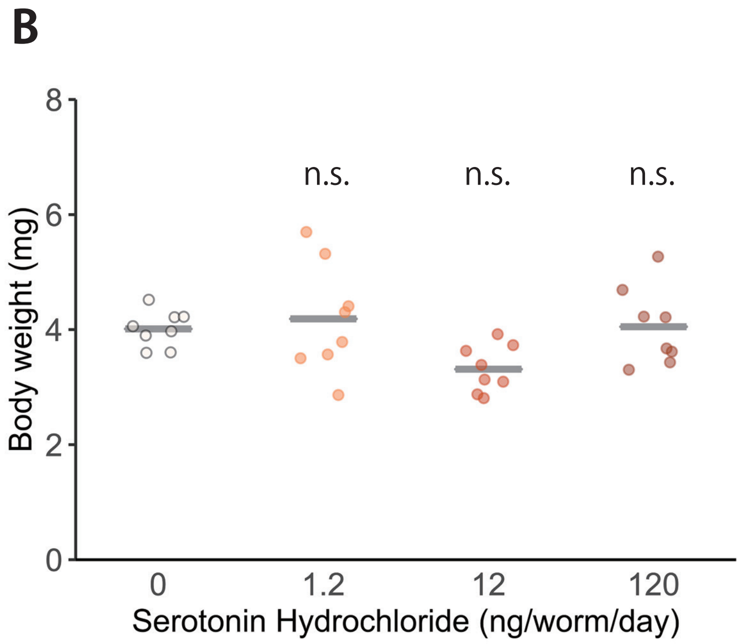

C

	Serotonin Hydrochloride (ng/worm/day)			
	0 (Control)	1.2	12	120
Fissioning rate	0 / 30	1 / 29	1 / 29	0 / 30

Supplementary Figure S5. Additional information on the worms in the serotonin feeding experiment. (A) The formation of supernumerary ovaries was observed in the same worm shown in Fig. 4D, which was fed a low serotonin concentration (1.2 ng/worm/d). Supernumerary ovaries are extra ectopic ovaries often found in experimentally sexualized worm of *D. ryukyuensis*. Domains bounded by the red line are the female germ cell masses (ovaries). Ov, ovary; sov, supernumerary ovary; in, intestine. (B) Body weights of the serotonin-fed worms were not significantly different from that in the control worms (Tukey's HSD test: n.s., not significant). (C) Fissioning rate of the control and serotonin-fed groups. Fissioning was rarely observed, being found only once in the group fed a low serotonin concentration (1.2 ng/worm/d) and once in the group fed an intermediate serotonin concentration (12 ng/worm/d) during the 4 weeks of the bioassay.

Supplementary Table S1. Summary of RNA-seq, de novo assembly, and mapping statistics

Sequencing statistics	Number of raw reads	228,246,618
	Number of reads per sample after quality-control filtering	15,216,441 ± 6,639,502
De novo assembly and mapping statistics	Total unigenes	132,884
	Total contigs	181,393
	N50 (bp)	1,063
	Median length (bp)	413
	Average length (bp)	718.5
	Total predicted CDSs	57,762
	Annotated CDSs	29,734
	Mapping rate (%)	96.5 ± 0.2

Supplementary Table S2. GO enrichment analysis of asexual and sexual DEGs. For the asexual DEGs, the bold font indicates pathways involved in neurological processes. For the sexual DEGs, the bold font indicates pathways involved in reproduction (DEG identification criteria: likelihood ratio test, FDR < 0.01).

Sexuality	Gene Ontology term (biological process)	Count	Fold enrichment	*P*-value	FDR
Asexual	**GO:0019226~transmission of nerve impulse**	10	33.9	1.80E-11	1.73E-08
	GO:0007267~cell-cell signaling	10	32.0	2.99E-11	1.43E-08
	**GO:0007268~synaptic transmission**	9	38.0	1.12E-10	3.59E-08
	**GO:0050877~neurological system process**	11	19.4	2.73E-10	6.53E-08
	GO:0048878~chemical homeostasis	9	19.6	2.04E-08	3.91E-06
	GO:0042592~homeostatic process	11	12.2	2.25E-08	3.60E-06
	GO:0042391~regulation of membrane potential	7	39.7	2.56E-08	3.50E-06
	GO:0006873~cellular ion homeostasis	7	21.9	8.51E-07	1.02E-04
	GO:0055082~cellular chemical homeostasis	7	21.7	8.83E-07	9.39E-05
	GO:0007610~behavior	7	20.1	1.39E-06	1.34E-04
	GO:0050801~ion homeostasis	7	19.6	1.62E-06	1.41E-04
	GO:0016192~vesicle-mediated transport	8	13.8	1.87E-06	1.50E-04
	**GO:0001505~regulation of neurotransmitter levels**	5	52.7	2.60E-06	1.91E-04
	GO:0007166~cell surface receptor linked signal transduction	8	9.3	2.39E-05	1.63E-03
	GO:0019725~cellular homeostasis	7	11.7	3.05E-05	1.94E-03
	**GO:0030182~neuron differentiation**	6	16.5	3.24E-05	1.94E-03
	GO:0032989~cellular component morphogenesis	6	16.0	3.69E-05	2.08E-03
	GO:0044057~regulation of system process	5	24.1	5.61E-05	2.98E-03
	**GO:0031175~neuron projection development**	5	22.6	7.27E-05	3.66E-03
	GO:0046903~secretion	5	20.9	9.72E-05	4.65E-03
	GO:0030534~adult behavior	4	41.3	1.27E-04	5.79E-03
	GO:0007155~cell adhesion	5	19.1	1.39E-04	6.05E-03
	GO:0019953~sexual reproduction	5	19.1	1.39E-04	6.05E-03
	GO:0022610~biological adhesion	5	19.0	1.41E-04	5.87E-03
	**GO:0048666~neuron development**	5	18.0	1.73E-04	6.88E-03
	GO:0030030~cell projection organization	5	17.3	2.01E-04	7.68E-03
	**GO:0007274~neuromuscular synaptic transmission**	3	133.7	2.28E-04	8.38E-03
Sexual	GO:0007049~cell cycle	34	11.5	2.02E-24	3.25E-21
	GO:0019941~modification-dependent protein catabolic process	31	11.4	3.65E-22	2.94E-19
	GO:0043632~modification-dependent macromolecule catabolic process	31	11.3	4.93E-22	2.65E-19
	GO:0051603~proteolysis involved in cellular protein catabolic process	32	10.6	5.80E-22	2.33E-19
	GO:0030163~protein catabolic process	33	10.0	6.32E-22	2.04E-19
	GO:0044257~cellular protein catabolic process	32	10.5	7.94E-22	2.13E-19
	GO:0045449~regulation of transcription	39	6.7	7.10E-20	1.63E-17
	GO:0009057~macromolecule catabolic process	41	5.9	5.56E-19	1.12E-16
	GO:0022403~cell cycle phase	23	13.3	8.00E-18	1.43E-15

Table. S2 (continued)

Sexual	GO:0006259~DNA metabolic process	40	5.4	3.21E-17	5.16E-15
	GO:0000279~M phase	21	14.9	3.43E-17	5.02E-15
	GO:0044265~cellular macromolecule catabolic process	37	5.9	3.61E-17	4.84E-15
	GO:0006350~transcription	36	6.1	3.64E-17	4.50E-15
	GO:0022402~cell cycle process	24	10.8	1.36E-16	1.28E-14
	GO:0006508~proteolysis	41	4.9	4.37E-16	4.76E-14
	GO:0043009~chordate embryonic development	17	13.3	3.59E-13	3.61E-11
	**GO:0006310~DNA recombination**	15	17.2	3.65E-13	3.46E-11
	GO:0009792~embryonic development ending in birth or egg hatching	17	12.4	1.07E-12	9.54E-11
	GO:0051276~chromosome organization	16	10.2	9.19E-11	7.79E-09
	GO:0006955~immune response	12	17.8	1.06E-10	8.55E-09
	GO:0033554~cellular response to stress	30	4.3	1.30E-10	9.99E-09
	GO:0006974~response to DNA damage stimulus	26	5.0	1.66E-10	1.22E-08
	GO:0009628~response to abiotic stimulus	16	9.5	2.40E-10	1.68E-08
	GO:0009314~response to radiation	13	13.6	3.24E-10	2.17E-08
	GO:0006281~DNA repair	25	5.0	3.52E-10	2.27E-08
	GO:0008202~steroid metabolic process	11	19.4	3.60E-10	2.23E-08
	GO:0006260~DNA replication	19	6.9	5.65E-10	3.37E-08
	GO:0001701~in utero embryonic development	12	15.0	6.77E-10	3.89E-08
	GO:0051301~cell division	14	11.0	6.88E-10	3.82E-08
	GO:0007242~intracellular signaling cascade	21	5.8	1.01E-09	5.44E-08
	GO:0006261~DNA-dependent DNA replication	12	13.9	1.49E-09	7.72E-08
	**GO:0048609~reproductive process in a multicellular organism**	13	11.7	1.81E-09	9.10E-08
	**GO:0032504~multicellular organism reproduction**	13	11.6	1.88E-09	9.18E-08
	GO:0002520~immune system development	12	13.5	2.11E-09	9.98E-08
	GO:0016071~mRNA metabolic process	21	5.5	2.67E-09	1.23E-07
	GO:0007067~mitosis	11	14.6	5.66E-09	2.53E-07
	GO:0000280~nuclear division	11	14.5	6.21E-09	2.70E-07
	GO:0000087~M phase of mitotic cell cycle	11	14.1	7.79E-09	3.30E-07
	GO:0048285~organelle fission	11	14.0	8.53E-09	3.52E-07
	**GO:0007276~gamete generation**	11	13.6	1.11E-08	4.46E-07
	GO:0000278~mitotic cell cycle	14	8.7	1.17E-08	4.60E-07
	**GO:0048232~male gamete generation**	9	18.5	3.70E-08	1.42E-06
	**GO:0007283~spermatogenesis**	9	18.5	3.70E-08	1.42E-06
	**GO:0019953~sexual reproduction**	11	11.7	4.59E-08	1.72E-06
	**GO:0051327~M phase of meiotic cell cycle**	9	17.6	5.51E-08	2.02E-06
	**GO:0007126~meiosis**	9	17.6	5.51E-08	2.02E-06
	GO:0006986~response to unfolded protein	7	33.6	6.86E-08	2.45E-06
	**GO:0051321~meiotic cell cycle**	9	17.0	7.23E-08	2.53E-06
	GO:0006397~mRNA processing	18	5.2	9.23E-08	3.16E-06
	GO:0051789~response to protein stimulus	8	21.9	9.44E-08	3.17E-06
	GO:0016125~sterol metabolic process	8	20.1	1.71E-07	5.61E-06
	GO:0042113~B cell activation	7	27.4	2.38E-07	7.67E-06
	GO:0002252~immune effector process	7	23.9	5.30E-07	1.67E-05
	GO:0009416~response to light stimulus	9	13.0	5.50E-07	1.70E-05
	GO:0046649~lymphocyte activation	8	16.2	7.32E-07	2.22E-05

Table. S2 (continued)

	GO:0006511~ubiquitin-dependent protein catabolic process	12	7.5	7.83E-07	2.33E-05
	GO:0016446~somatic hypermutation of immunoglobulin genes	5	68.4	7.96E-07	2.33E-05
Sexual	GO:0002566~somatic diversification of immune receptors via somatic mutation	5	68.4	7.96E-07	2.33E-05
	GO:0042981~regulation of apoptosis	14	5.9	9.10E-07	2.62E-05
	GO:0043067~regulation of programmed cell death	14	5.9	9.78E-07	2.76E-05
	GO:0010941~regulation of cell death	14	5.9	9.94E-07	2.76E-05
	GO:0002440~production of molecular mediator of immune response	6	33.5	1.03E-06	2.82E-05
	**GO:0007140~male meiosis**	5	62.2	1.20E-06	3.21E-05
	GO:0006952~defense response	8	14.6	1.49E-06	3.93E-05
	GO:0045321~leukocyte activation	8	14.4	1.62E-06	4.21E-05
	GO:0008203~cholesterol metabolic process	7	19.7	1.65E-06	4.23E-05
	GO:0009411~response to UV	7	19.5	1.76E-06	4.42E-05
	GO:0006302~double-strand break repair	8	14.0	1.93E-06	4.78E-05
	GO:0001775~cell activation	8	13.4	2.69E-06	6.56E-05
	GO:0010604~positive regulation of macromolecule metabolic process	13	5.9	2.91E-06	7.00E-05
	GO:0006298~mismatch repair	6	25.7	3.95E-06	9.35E-05
	GO:0030097~hemopoiesis	8	11.6	6.60E-06	1.54E-04
	**GO:0007127~meiosis I**	6	22.8	7.08E-06	1.63E-04
	GO:0006270~DNA replication initiation	7	15.2	7.54E-06	1.71E-04
	GO:0045941~positive regulation of transcription	10	7.4	1.00E-05	2.24E-04
	GO:0010628~positive regulation of gene expression	10	7.4	1.03E-05	2.26E-04
	**GO:0048608~reproductive structure development**	7	14.2	1.12E-05	2.43E-04
	GO:0048534~hemopoietic or lymphoid organ development	8	10.5	1.27E-05	2.73E-04
	**GO:0008406~gonad development**	6	19.1	1.69E-05	3.58E-04
	GO:0006268~DNA unwinding during replication	5	31.1	2.08E-05	4.35E-04
	GO:0016447~somatic recombination of immunoglobulin gene segments	5	31.1	2.08E-05	4.35E-04
	GO:0010033~response to organic substance	12	5.3	2.20E-05	4.55E-04
	GO:0032446~protein modification by small protein conjugation	8	9.6	2.30E-05	4.68E-04
	GO:0002449~lymphocyte mediated immunity	5	29.7	2.49E-05	5.01E-04
	GO:0002377~immunoglobulin production	5	29.1	2.71E-05	5.39E-04
	GO:0016445~somatic diversification of immunoglobulins	5	29.1	2.71E-05	5.39E-04
	GO:0010557~positive regulation of macromolecule biosynthetic process	10	6.5	2.77E-05	5.44E-04
	GO:0045935~positive regulation of nucleobase, nucleoside, nucleotide and nucleic acid metabolic process	10	6.5	2.77E-05	5.44E-04
	GO:0051174~regulation of phosphorus metabolic process	9	7.5	3.13E-05	6.06E-04
	GO:0019220~regulation of phosphate metabolic process	9	7.5	3.13E-05	6.06E-04
	GO:0032508~DNA duplex unwinding	5	27.9	3.20E-05	6.14E-04

Table. S2 (continued)

	GO:0051173~positive regulation of nitrogen compound metabolic process	10	6.4	3.25E-05	6.16E-04
	GO:0016444~somatic cell DNA recombination	5	27.4	3.47E-05	6.50E-04
Sexual	GO:0002562~somatic diversification of immune receptors via germline recombination within a single locus	5	27.4	3.47E-05	6.50E-04
	GO:0031328~positive regulation of cellular biosynthetic process	10	6.3	3.62E-05	6.70E-04
	**GO:0045137~development of primary sexual characteristics**	6	16.3	3.68E-05	6.72E-04
	GO:0065003~macromolecular complex assembly	13	4.5	3.92E-05	7.10E-04
	GO:0032392~DNA geometric change	5	26.3	4.06E-05	7.26E-04
	GO:0016265~death	10	6.2	4.10E-05	7.26E-04
	GO:0009891~positive regulation of biosynthetic process	10	6.2	4.32E-05	7.56E-04
	GO:0070647~protein modification by small protein conjugation or removal	8	8.7	4.34E-05	7.51E-04
	GO:0002250~adaptive immune response	5	25.8	4.38E-05	7.49E-04
	GO:0002200~somatic diversification of immune receptors	5	25.8	4.38E-05	7.49E-04
	GO:0002460~adaptive immune response based on somatic recombination of immune receptors built from immunoglobulin superfamily domains	5	25.8	4.38E-05	7.49E-04
	GO:0051726~regulation of cell cycle	9	7.1	4.82E-05	8.17E-04
	GO:0008283~cell proliferation	8	8.5	4.90E-05	8.22E-04
	GO:0016567~protein ubiquitination	7	10.2	6.97E-05	1.16E-03
	GO:0002443~leukocyte mediated immunity	5	22.4	7.62E-05	1.25E-03
	**GO:0007548~sex differentiation**	6	13.7	8.36E-05	1.36E-03
	GO:0043933~macromolecular complex subunit organization	16	3.4	8.83E-05	1.42E-03
	GO:0006915~apoptosis	9	6.5	8.87E-05	1.41E-03
	GO:0010605~negative regulation of macromolecule metabolic process	11	4.9	9.45E-05	1.49E-03
	GO:0012501~programmed cell death	9	6.3	1.04E-04	1.63E-03
	GO:0006468~protein amino acid phosphorylation	13	4.1	1.07E-04	1.66E-03
	GO:0042592~homeostatic process	13	4.0	1.16E-04	1.78E-03
	GO:0006325~chromatin organization	7	9.3	1.18E-04	1.80E-03
	GO:0006694~steroid biosynthetic process	5	19.8	1.23E-04	1.86E-03
	GO:0009100~glycoprotein metabolic process	8	7.3	1.29E-04	1.92E-03
	GO:0035295~tube development	7	9.1	1.31E-04	1.94E-03
	GO:0030183~B cell differentiation	4	39.1	1.46E-04	2.14E-03
	GO:0051252~regulation of RNA metabolic process	14	3.6	1.71E-04	2.48E-03
	GO:0043414~biopolymer methylation	5	17.8	1.89E-04	2.71E-03
	GO:0032259~methylation	5	17.8	1.89E-04	2.71E-03
	GO:0035239~tube morphogenesis	6	11.5	1.90E-04	2.71E-03
	GO:0042770~DNA damage response, signal transduction	5	17.5	1.99E-04	2.80E-03
	GO:0010212~response to ionizing radiation	5	17.5	1.99E-04	2.80E-03
	GO:0008219~cell death	9	5.7	2.18E-04	3.05E-03
	GO:0050877~neurological system process	10	4.9	2.31E-04	3.21E-03

Table. S2 (continued)

Sexual	GO:0002204~somatic recombination of immunoglobulin genes during immune response	4	33.2	2.40E-04	3.30E-03
	GO:0002208~somatic diversification of immunoglobulins during immune response	4	33.2	2.40E-04	3.30E-03
	GO:0045190~isotype switching	4	33.2	2.40E-04	3.30E-03
	GO:0002381~immunoglobulin production during immune response	4	33.2	2.40E-04	3.30E-03
	GO:0008104~protein localization	20	2.6	2.61E-04	3.56E-03
	GO:0044093~positive regulation of molecular function	9	5.5	2.74E-04	3.71E-03
	GO:0045934~negative regulation of nucleobase, nucleoside, nucleotide and nucleic acid metabolic process	8	6.4	2.95E-04	3.94E-03
	GO:0035264~multicellular organism growth	4	30.4	3.12E-04	4.14E-03
	GO:0051172~negative regulation of nitrogen compound metabolic process	8	6.2	3.33E-04	4.38E-03
	GO:0040007~growth	6	9.7	4.11E-04	5.36E-03
	**GO:0003006~reproductive developmental process**	7	7.3	4.40E-04	5.69E-03
	GO:0016568~chromatin modification	6	9.5	4.45E-04	5.71E-03
	GO:0002521~leukocyte differentiation	5	14.0	4.75E-04	6.05E-03
	GO:0007346~regulation of mitotic cell cycle	6	9.4	4.81E-04	6.08E-03
	GO:0006355~regulation of transcription, DNA-dependent	13	3.4	4.98E-04	6.24E-03
	GO:0007059~chromosome segregation	5	13.7	5.13E-04	6.38E-03
	GO:0016064~immunoglobulin mediated immune response	4	25.5	5.29E-04	6.53E-03
	GO:0019724~B cell mediated immunity	4	25.5	5.29E-04	6.53E-03
	**GO:0007131~reciprocal meiotic recombination**	4	24.9	5.66E-04	6.94E-03
	GO:0006413~translational initiation	6	9.0	5.75E-04	6.99E-03
	GO:0035148~tube lumen formation	4	22.8	7.32E-04	8.82E-03
	GO:0055092~sterol homeostasis	4	22.3	7.77E-04	9.30E-03
	GO:0042632~cholesterol homeostasis	4	22.3	7.77E-04	9.30E-03
	GO:0051053~negative regulation of DNA metabolic process	4	21.9	8.25E-04	9.79E-03
	GO:0000209~protein polyubiquitination	4	21.9	8.25E-04	9.79E-03
	GO:0000724~double-strand break repair via homologous recombination	4	21.9	8.25E-04	9.79E-03

Supplementary Table S3. DEGs in the enriched KEGG pathways. Pathways indicated in bold represent amino acid metabolism pathways (DEG identification criteria: likelihood ratio test, FDR < 0.01).

Sexuality	Pathway	Count	KEGG gene name
Asexual	**mmu00260:Glycine, serine and threonine metabolism**	6	CTH, DAO, DLD, DMGDH, PHGDH, TDH
	**mmu00350:Tyrosine metabolism**	5	DDC, GOT1, HGD, HPD, TYR
	**mmu00380:Tryptophan metabolism**	4	ACMSD, DDC, KMO, TDO2
	**mmu00330:Arginine and proline metabolism**	4	ACY1, CPS1, DAO, GOT1
	mmu04080:Neuroactive ligand-receptor interaction	4	GLRA2, GLRB, GRIK4, GRIN2B
	mmu04810:Regulation of actin cytoskeleton	4	GSN, ITGAV, MYLK, WAS
	mmu04510:Focal adhesion	4	CAV3, ITGAV, MYLK, ZYX
	**mmu00360:Phenylalanine metabolism**	3	DDC, GOT1, HPD
	mmu04514:Cell adhesion molecules (CAMs)	3	ITGAV, L1CAM, SDC2
	mmu00620:Pyruvate metabolism	3	DLD, GRHPR, LDHD
	**mmu00270:Cysteine and methionine metabolism**	3	CDO1, CTH, GOT1
	mmu05200:Pathways in cancer	4	AXIN2, FZD4, ITGAV, WNT4
	mmu05217:Basal cell carcinoma	3	AXIN2, FZD4, WNT4
	**mmu00250:Alanine, aspartate and glutamate metabolism**	3	CPS1, GOT1, GPT
	mmu00564:Glycerophospholipid metabolism	3	ACHE, CHAT, LCAT
	mmu04666:Fc gamma R-mediated phagocytosis	3	GSN, PRKCE, WAS
	mmu04916:Melanogenesis	3	FZD4, TYR, WNT4
	mmu04270:Vascular smooth muscle contraction	3	KCNMA1, MYLK, PRKCE
	mmu04310:Wnt signaling pathway	3	AXIN2, FZD4, WNT4
Sexual	mmu04120:Ubiquitin mediated proteolysis	22	CBL, CDC16, CDC27, CUL2, CUL3, HERC2, HERC4, PIAS1, PPIL2, RBX1, TCEB1, TRIM37, UBA1, UBE2A, UBE2H, UBE2I, UBE2J1, UBE2M, UBE2O, UBE2S, UBE4A, UBE4B
	mmu00230:Purine metabolism	18	ADA, ADCY1, ADCY2, ADCY5, ADK, GMPS, PAICS, PDE6D, PFAS, POLA1, POLD1, POLD2, POLE2, POLR1B, POLR2E, POLR3B, PRUNE, RRM1
	mmu04110:Cell cycle	16	CDC16, CDC25C, CDC27, CDC7, CDK1, MCM2, MCM3, MCM4, MCM5, MCM6, MCM7, PCNA, PLK1, RBX1, TTK, WEE1
	mmu05200:Pathways in cancer	19	APC2, ARNT, BCL2L1, BRCA2, CASP8, CBL, CCDC6, CDH1, CUL2, MLH1, MSH2, MSH6, PIAS1, RAD51, RBX1, SLC2A1, SUFU, TCEB1, TRAF3
	mmu03030:DNA replication	13	MCM2, MCM3, MCM4, MCM5, MCM6, MCM7, PCNA, POLA1, POLD1, POLD2, POLE2, RFC1, RPA2
	mmu03430:Mismatch repair	11	EXO1, MLH1, MLH3, MSH2, MSH6, PCNA, PMS2, POLD1, POLD2, RFC1, RPA2
	mmu04114:Oocyte meiosis	10	ADCY1, ADCY2, ADCY5, AURKA, CDC16, CDC25C, CDC27, CDK1, PLK1, RBX1
	mmu03440:Homologous recombination	9	BRCA2, EME1, POLD1, POLD2, RAD51, RAD51C, RAD54B, RAD54L, RPA2
	mmu04914:Progesterone-mediated oocyte maturation	8	ADCY1, ADCY2, ADCY5, CDC16, CDC25C, CDC27, CDK1, PLK1
	mmu03420:Nucleotide excision repair	9	ERCC3, ERCC5, PCNA, POLD1, POLD2, POLE2, RBX1, RFC1, RPA2
	mmu00240:Pyrimidine metabolism	10	CAD, CDA, POLA1, POLD1, POLD2, POLE2, POLR1B, POLR2E, POLR3B, RRM1
	**mmu00380:Tryptophan metabolism**	7	ABP1, ALDH9A1, CAT, DDC, HADHA, KYNU, WARS
	mmu03018:RNA degradation	8	CNOT3, CNOT4, DCP2, DDX6, PATL1, SKIV2L, WDR61, XRN2
	mmu00052:Galactose metabolism	6	G6PC, GAA, GALE, GALT, PGM2, UGP2
	mmu00970:Aminoacyl-tRNA biosynthesis	7	FARSA, FARSB, HARS, PSTK, RARS, VARS, WARS

Table. S3 (continued)

Sexual	mmu04612:Antigen processing and presentation	6	CTSB, HSPA5, NFYA, NFYB, NFYC, PSME3
	mmu03040:Spliceosome	9	CDC5L, DDX42, DDX5, DHX15, PRPF31, PRPF4, PRPF6, SNRPA, U2AF2
	mmu00520:Amino sugar and nucleotide sugar metabolism	6	AMDHD2, GALE, GALT, PGM2, PGM3, UGP2
	mmu04622:RIG-I-like receptor signaling pathway	5	CASP8, DDX3X, IFIH1, OTUD5, TRAF3
	mmu03410:Base excision repair	5	APEX1, PCNA, POLD1, POLD2, POLE2
	mmu00330:Arginine and proline metabolism	5	ABP1, ACY1, ALDH9A1, AMD1, SRM
	mmu05211:Renal cell carcinoma	5	ARNT, CUL2, RBX1, SLC2A1, TCEB1
	mmu00510:N-Glycan biosynthesis	5	ALG1, ALG2, ALG6, GANAB, RPN2
	mmu04144:Endocytosis	6	CBL, CHMP2A, HGS, LDLR, LDLRAP1, USP8
	mmu00340:Histidine metabolism	4	ABP1, ALDH9A1, DDC, HAL
	mmu00500:Starch and sucrose metabolism	4	G6PC, GAA, PGM2, UGP2
	mmu04910:Insulin signaling pathway	5	CBL, EIF4E, EIF4EBP1, ELK1, G6PC
	mmu04310:Wnt signaling pathway	5	APC2, CACYBP, CSNK2B, RBX1, RUVBL1
	mmu03050:Proteasome	5	PSMC5, PSMC6, PSMD1, PSMD4, PSME3
	mmu00010:Glycolysis / Gluconeogenesis	4	ALDH9A1, G6PC, GAPDH, PGM2
	mmu04142:Lysosome	5	CTSB, CTSC, GAA, HGSNAT, NAGA
	mmu02010:ABC transporters	4	ABCA5, ABCC4, ABCD2, ABCD3
	mmu04540:Gap junction	4	ADCY1, ADCY2, ADCY5, CDK1
	mmu05213:Endometrial cancer	4	APC2, CDH1, ELK1, MLH1
	mmu04916:Melanogenesis	4	ADCY1, ADCY2, ADCY5, TYR
	mmu05210:Colorectal cancer	4	APC2, MLH1, MSH2, MSH6
	mmu04912:GnRH signaling pathway	4	ADCY1, ADCY2, ADCY5, ELK1
	mmu01040:Biosynthesis of unsaturated fatty acids	3	HADHA, HSD17B12, YOD1
	mmu04062:Chemokine signaling pathway	4	ADCY1, ADCY2, ADCY5, PXN
	mmu00903:Limonene and pinene degradation	3	ALDH9A1, HADHA, YOD1
	mmu05340:Primary immunodeficiency	3	ADA, BTK, DCLRE1C
	mmu04020:Calcium signaling pathway	4	ADCY1, ADCY2, VDAC1, VDAC2
	mmu05016:Huntington's disease	5	CASP8, IFT57, POLR2E, VDAC1, VDAC2
	mmu00360:Phenylalanine metabolism	3	DDC, PAH, PRDX6
	mmu04115:p53 signaling pathway	3	CASP8, CDK1, PPM1D
	mmu00983:Drug metabolism	3	CDA, GMPS, TPMT
	mmu04810:Regulation of actin cytoskeleton	4	APC2, ENAH, IQGAP1, PXN
	mmu00410:beta-Alanine metabolism	3	ALDH9A1, HADHA, SRM
	mmu00620:Pyruvate metabolism	3	ALDH9A1, MDH1, ME2
	mmu04630:Jak-STAT signaling pathway	3	BCL2L1, CBL, PIAS1
	mmu00640:Propanoate metabolism	3	ALDH9A1, HADHA, PCCA
	mmu05212:Pancreatic cancer	3	BCL2L1, BRCA2, RAD51
	mmu05012:Parkinson's disease	4	UBA1, UBE2J1, VDAC1, VDAC2
	mmu00480:Glutathione metabolism	3	GSR, RRM1, SRM
	mmu05014:Amyotrophic lateral sclerosis (ALS)	3	BCL2L1, CAT, DERL1
	mmu05414:Dilated cardiomyopathy	3	ADCY1, ADCY2, ADCY5
	mmu03022:Basal transcription factors	3	GTF2B, GTF2F1, TAF5
	mmu03020:RNA polymerase	3	POLR1B, POLR2E, POLR3B
	mmu05222:Small cell lung cancer	3	BCL2L1, PIAS1, TRAF3
	mmu04012:ErbB signaling pathway	3	CBL, EIF4EBP1, ELK1
	mmu00260:Glycine, serine and threonine metabolism	3	AGXT2, GNMT, PSPH
	mmu00280:Valine, leucine and isoleucine degradation	3	ALDH9A1, HADHA, PCCA

Supplementary Table S4. Detailed information on DEGs in three amino acid metabolic pathways enriched both in asexual and sexual worms: tryptophan metabolism; glycine, serine, and threonine metabolism; and arginine and proline metabolism. All 31 DEGs were chosen for validation by qRT-PCR. Primer sets used for qRT-PCR are also indicated. (DEG identification criteria: likelihood ratio test, FDR < 0.01).

Pathway	DEG	Contig ID	logFC	logCPM	Likelihood ratio	*P*-value	FDR	KEGG orthology ID	KEGG gene name	Forward primer (5′ -3′)	Reverse primer (5′ -3′)
mmu00380:Tryptophan metabolism	Asexual	TR47433|c3_g1_i7	1.32E+00	4.73E+00	3.30E+01	9.16E-09	2.77E-07	K00453	TDO2	ATGCCGTTCTGCGATTTATG	CCATTTGACTAAGCCTACGAAGC
		TR20794|c0_g2_i1	1.65E+00	3.67E+00	1.45E+01	1.38E-04	2.48E-03	K03392	ACMSD	AATTCTGGCTGCCTTGGTTG	CCATCGTGTATGGAAATGATCC
		TR48106|c0_g2_i5	1.99E+00	9.51E-01	1.43E+01	1.55E-04	2.76E-03	K01593	DDC	CCGTCTGAAGGGAAACAATGAG	TTCCGGGTCACAGAATGAAG
		TR39289|c0_g1_i1	6.10E-01	5.40E+00	1.43E+01	1.57E-04	2.78E-03	K00486	KMO	TTCTAGCGTACATCGGAATCG	TCCCATCGAGGAAGAGAAGC
		TR44018|c0_g1_i1	6.79E-01	6.18E+00	1.18E+01	5.86E-04	9.11E-03	K00486	KMO	AATCGCTTTGGCATCTCTATTTC	TGGTCAAGGAATGAATTGTGG
	Sexual	TR33768|c0_g1_i1	-4.58E+00	5.74E+00	2.95E+02	4.26E-66	6.13E-64	K07515	HADHA	GCCTGGTCTGAAGAATGCAAC	TCAAACCTTCGGCTTTAGGG
		TR35961|c1_g1_i1	-4.95E+00	6.30E+00	1.47E+02	8.85E-34	7.02E-32	K01593	DDC	TGATGCTATCGGATGCGTTG	ATGACTCCTCCTCCACCAAGAC
		TR41896|c0_g1_i2	-5.61E+00	2.70E+00	1.27E+02	2.23E-29	1.57E-27	K03781	CAT	CATGTGGCGAAATCCATCTG	TGCGTGATCCCAATATGATG
		TR35961|c1_g1_i2	-5.25E+00	3.82E+00	7.21E+01	2.10E-17	1.01E-15	K01593	DDC	TGATGCTATCGGATGCGTTG	ATGACTCCTCCTCCACCAAGAC
		TR38793|c0_g1_i1	-1.41E+00	5.03E+00	5.72E+01	3.89E-14	1.64E-12	K00149	ALDH9A1	TACGACCAGTTGCCACACTACC	TCCCGAAGGATTGGTTTCTG
		TR47755|c3_g3_i3	-4.46E+00	1.39E+00	3.82E+01	6.44E-10	2.12E-08	K11182	ABP1	TCTCCAGGTGTTGATTGTCCAG	TGTCTGCCTAATGGTTTAGTGCTC
		TR29012|c0_g1_i1	-1.96E+00	5.23E+00	2.96E+01	5.33E-08	1.51E-06	K01556	KYNU	TTTATCGAAACAAACGGCAAAG	GCGTGTGCTAAATCCCAACC
		TR42512|c1_g3_i1	-1.01E+00	3.66E+00	1.87E+01	1.50E-05	3.17E-04	K01867	WARS	GCAGCATCTTTAATAGCCTGTGG	TGTGGCATTGTTTGAACTCG
mmu00260:Glycine, serine and threonine metabolism	Asexual	TR8800|c0_g1_i1	1.51E+00	5.52E+00	5.35E+01	2.64E-13	1.06E-11	K00273	DAO	TCGCTACCAATCCAAAGCATC	CGGCTGTTTCCCACAATTTC
		TR45562|c7_g2_i1	1.56E+00	3.22E+00	2.70E+01	2.08E-07	5.57E-06	K00102	DLD	TTGGCAAGAGGCTGCAATAC	TTGGAGTGACCAGATTCAAGTG
		TR36760|c0_g1_i1	1.05E+00	4.50E+00	2.33E+01	1.37E-06	3.32E-05	K15789	TDH	TTTCATCGAACAGTCCACCTG	AATTCCTTTGAGGCAGTCGTC
		TR43829|c0_g1_i1	1.10E+00	4.23E+00	2.23E+01	2.30E-06	5.42E-05	K00058	PHGDH	CATTAACTATACCGCCTCTTGCAC	TCCTTTGATTCCGCAAACG
		TR44631|c2_g1_i2	8.39E-01	5.21E+00	1.74E+01	3.04E-05	6.12E-04	K01758	CTH	ACGGCCATTCAGATGTGTTG	GAGTACGCAATCCACGCAAG
		TR41695|c0_g1_i1	6.63E-01	5.97E+00	1.69E+01	3.92E-05	7.75E-04	K00315	DMGDH	ACATTCACGCTCCATTGCTG	TGTTGCTGGGTGGGAGAAAC
	Sexual	TR43837|c0_g1_i1	-1.73E+00	6.17E+00	8.81E+01	6.20E-21	3.39E-19	K00827	AGXT2	TCAGTTGGGCATTGTCATCC	GGCGAACTCGTGAATTTCTG
		TR41013|c0_g1_i1	-1.39E+00	4.81E+00	3.11E+01	2.51E-08	7.30E-07	K01079	PSPH	TGATTGGTGATGGTGTTACCG	TCGTCGTTTGACTTCTTCTCG
		TR25277|c1_g1_i1	-7.83E-01	6.76E+00	2.20E+01	2.77E-06	6.47E-05	K00552	GNMT	GCATAACTGAATCAACTCCAGTCC	GGGATCGAAACAAGAAAGAACG
mmu00330:Arginine and proline metabolism	Asexual	TR8800|c0_g1_i1	1.51E+00	5.52E+00	5.35E+01	2.64E-13	1.06E-11	K00273	DAO	TCGCTACCAATCCAAAGCATC	CGGCTGTTTCCCACAATTTC
		TR44264|c2_g1_i1	1.06E+00	5.27E+00	3.29E+01	9.51E-09	2.87E-07	K01948	CPS1	TCGCCTTCTGCATTTGTTTG	TGGTCAGGCTGGTGAATTTG
		TR39506|c0_g1_i3	2.78E+00	1.64E+00	3.05E+01	3.37E-08	9.72E-07	K14677	ACY1	GATTTGGAATACCTTCATCCATAACG	TATTTGTTCCAGACGAAGAAGTCG
		TR45543|c0_g1_i1	5.85E-01	6.72E+00	1.26E+01	3.85E-04	6.26E-03	K14454	GOT1	GCAACCGTCCTTAGCAATCC	CATGTTCCCATGAACCAGGAG
	Sexual	TR28661|c0_g1_i1	-1.18E+01	6.11E+00	7.87E+02	3.62E-173	1.73E-169	K14677	ACY1	GCAGCATTTGAATCGGTTTG	TTGCGAACGAATCTGCTGTC
		TR47390|c0_g2_i1	-1.95E+00	4.29E+00	6.40E+01	1.24E-15	5.58E-14	K00797	SRM	GGTAGTTCTTGTTCGATGGGTTC	CAGTATGCAAGTGGTTGTGTTCC
		TR38793|c0_g1_i1	-1.41E+00	5.03E+00	5.72E+01	3.89E-14	1.64E-12	K00149	ALDH9A1	TACGACCAGTTGCCACACTACC	TCCCGAAGGATTGGTTTCTG
		TR47755|c3_g3_i3	-4.46E+00	1.39E+00	3.82E+01	6.44E-10	2.12E-08	K11182	ABP1	TCTCCAGGTGTTGATTGTCCAG	TGTCTGCCTAATGGTTTAGTGCTC
		TR31601|c0_g1_i1	-6.83E-01	6.75E+00	1.73E+01	3.25E-05	6.52E-04	K01611	AMD1	CGATGCGTGGTTTCAGATTTAG	TATAGTGGGCGGGTAACTGTCG

Supplementary Table S5. Primer sets used for the synthesis of whole-mount *in situ* hybridization probes

	ID	Forward primer (5′ -3′)	Reverse primer (5′ -3′)
Asexual DEGs	TR47548	TTCGTGTTTGAGGCGTTCC	GAAGTTGCCGAGAATCCACAG
	TR46930	TGCTGCTGTTGTTGTTGCTG	TGGGCTGGTCAAATGAAAGAC
	TR46247	TGGACGGACAAAGATAATCGAG	CATGACTGTGATATTGGGCAAC
	TR46543	CGGCTGGATAATAAATGGGTTG	CAAGCGTTCAAGAGTTTCACAG
	TR43664	GAGAAGTGCATCGGTTTTATCAAC	AAAATTACGGTCTCCAAGAGCAAC
	TR30587	TTGTTCGGTGATTGACTGTCG	CGCACGCAATTTCTGTATTG
	TR49031	TCTCCATCATCCAATCATCGTC	CTGCCGGTCAAGATTCCTTC
Sexual DEGs	TR16580	GTTGACGCTTGCACCCTTTG	GCTTATCAAATGCCGTATAGCAACC
	TR34002	TTGAGGACTGTGAGGTGCTTG	TGCCGACTGCTTCTTAGTTCC
	TR37793	CAGAGGCACATAAACCCATTTC	TGCTGAAGACGCAAACACATC
	TR27538	GCTAATCGCTGCCAAACTGC	TTACCCATTCATCCTTCACCTC
	TR25120	TGGACTTGGTGGAGGAACTG	GCTAAGAATTGCTCATGGACACG
	TR67403	TTTAAATTTCTAAAATGAATCATCC	TATTTGATCACAATGGTTTGAC
	TR46108	TTTGCCTTTGTACGCCACAG	TTTGGCATTGTCGTTTCTGG
	TR11261	AAATCCAACACATCAGTTAATTCC	TCTCTCACTGCAGGTAATGTAGTC
	TR79134	CGACCGATAACAATAATGGAACG	AACATCAGCTTGAGCAATTCG
	TR45012	CGAGTTGTTTGAGCCAGAGC	CGAGAGGTCATTTCACTGTTGG
	TR30019	CATTGGACGTTGACCGTTTG	GCGCAAATTATCGCTGCTG
	TR28264	GTGGTAGGTCATGTTCGATTTCAG	TCATTCACGTTGGCGAATTG
	TR5825	ACACAGCATGCACACAAGC	TTGGTTGACAGACCGAATATC
	TR4019	GATTCTTGCGGGTCTAAGTGC	GGGTCATTTGTGGGTTTCAG
	TR3302	GCCGCAGAGTTGTTAGGAAAG	GGACTGGGATTTGGATTTGG
	TR28851	CGATTTCCCAAGTTTCTCTGATG	TCCTTTATCAATTGCGGTAG
	TR20914	CCCGCAAGAATCAGTTCCTC	GGGTAGCATAAGCGTGAAATGTG
	TR73547	CAAGTGGCTTATTGAAAGATTCTCG	TCCACCATTGCTTGACATCC
	TR39624	CAACAATTTTGCCGGATTTA	ATTTTTAGAAATATTCAATGGTGCATT
	TR49670	TTGAAATGTCTCCAGCAAAAACTA	AAAATCGAGATGTAGAACCAATCA
	TR20240	TGTCTCTAACAAGAATGGTCGAAT	AAATCGAGATGTAGATCCAATCAA

Supplementary Table S6. Primer sets used for qRT-PCR to validate the genes from the top 25 asexual DEGs and top 40 sexual DEGs with the largest log_2FC.

	ID	Forward primer (5′ -3′)	Reverse primer (5′ -3′)
Asexual DEGs	TR47548	ATTCGTGTTTGAGGCGTTCC	TTGGAGACCCAGGAGAAGTAGTG
	TR46930	TGGCACCGGACATTTGAAG	GCAACAACAACAGCAGCAATC
	TR46247	GACAATGTTACTGGACCTTTGGTG	CATGACTGTGATATTGGGCAAC
	TR46543	GCTGTCGAAACCAAACTTCACC	GAGTTCCTCTCAAGCGTTCAAG
	TR43664	AAGGCTGCTGTGAAATGTCG	TTGGAGGAAGCAAATGATCG
	TR30587	GGAAGATCGACACTTTAGGCATTC	CGCACGCAATTTCTGTATTGG
	TR49031	TCTCCATCATCCAATCATCGTC	TTTCGACAGACCGATTCTTCC
Sexual DEGs	TR16580	TTGCTCGATTACTGGAAGTTGC	CATCCAAAACTGTAACGCATGG
	TR34002	CAGGACGGCAGGAAAGAAATAG	TCCATCAAGCACCTCACAGTC
	TR37793	CGGTCCATTCCAAATAATCG	TGCTGAAGACGCAAACACATC
	TR27538	TCTCGATCGATACCATGTTCAG	GCGCGAAATCCTATCCCTTC
	TR25120	AAATGGGTCAATGCGGAAAC	CGCCTTCATAAACTCCTGTCG
	TR67403	CATCAGTTCATTGGGATGATCG	ATAAATTTCCGCGGGACCAG
	TR46108	CCGTAAGCAATTCCCATTCC	AACTCGGTTCACCAATAACTGC
	TR11261	GGGGAAAGATGAATGGGAAC	TGTTCAAGATGTTACTCAGCATTCG
	TR79134	CTGTCATTTCTATCGGGAATGC	ACGGTGATTTTTCAGCATCG
	TR45012	TCCTTCTTCCATCCCTTCTCC	GCTCTGGCTCAAACAACTCG
	TR30019	GCAAGAGCGTCATTACAGTTTCTC	CAAACGGTCAACGTCCAATG
	TR28264	CCAAAATCCAAAAGCGGAAG	GGTCGTACAATACTCGGCTGTG
	TR5825	CGACCACAATTCGGTTCAG	CACCGTTTATTTGTGGAGGAAG
	TR4019	GGGATCTGAAACCCACAAATG	CCGGGAATGATGTACGTTAGAAG
	TR3302	TGGTGGATGGACAGATGGAG	GCAACCAGCGTAAACCCTTC
	TR28851	CCAGCTTTTCCGTATTTTCTAAACC	TCCAGACTGGCTGATCAATAAAG
	TR20914	ATCGTTGGATTCAGCAAAGG	CTCCATTATGACTTGCATCTAGCC
	TR73547	CAAGTGGCTTATTGAAAGATTCTCG	TTCAAATCTGGGAATCCTCTTACAC
	TR39624	CCACAACTACTGTTATTCCATC	TTCCAACAATTGCAACG
	TR49670	GCGAAGTGAAGATGATGTGACTC	TTCTTCGTCTCGTCGTGTCC
	TR20240	GGAAAAGGATGTCCATCAAGC	TCGATCCTCCAAAAATGCTG
