# Supplementary material for: Transcriptomic analysis reveals differences in the regulation of amino acid metabolism in asexual and sexual planarians
Source: Sci Rep. 2019 Apr 16;9:6132. doi: 10.1038/s41598-019-42025-z (PMC6467871; doi:10.1038/s41598-019-42025-z)
Supplement: Supplementary file 3 — Permission [file 41598_2019_42025_MOESM3_ESM.pdf]

KEGG Database Project
Kanehisa Laboratories
www.kanehisa.jp

Ref: 180364

Permission is granted to Scientific Reports of Springer Nature Ltd to publish both in print and digital under the CC BY 4.0 open access license the following KEGG pathway map images in the article "Transcriptomic analysis reveals differences in the regulation of amino acid metabolism in asexual and sexual planarians" written by Kazuya Kobayashi and colleagues:

- Tryptophan metabolism (map00380)
- Glycine, serine and threonine metabolism (map00260)
- Arginine and proline metabolism (map00330)

subject to the condition that the original source is acknowledged by citing at least one KEGG paper.

Permission granted: Miwako Matsumoto

Miwako Matsumoto, Kanehisa Laboratories

Date: 10 December 2018

Copyright holder: Kanehisa Laboratories
